# Quantification of primary mitral regurgitation by echocardiography: A practical appraisal

**DOI:** 10.3389/fcvm.2023.1107724

**Published:** 2023-03-10

**Authors:** Alexandre Altes, Emmanuelle Vermes, Franck Levy, David Vancraeynest, Agnès Pasquet, André Vincentelli, Bernhard L. Gerber, Christophe Tribouilloy, Sylvestre Maréchaux

**Affiliations:** ^1^ GCS-Groupement des Hôpitaux de l’Institut Catholique de Lille/Lille Catholic Hospitals, Heart Valve Center, Cardiology Department, ETHICS EA 7446, Lille Catholic University, Lille, France; ^2^ Division of Cardiology, Department of Cardiovascular Diseases, Cliniques Universitaires St. Luc, Pôle de Recherche Cardiovasculaire (CARD), Institut de Recherche Expérimentale et Clinique (IREC), Université Catholique de Louvain, Brussels, Belgium; ^3^ UR UPJV 7517, Jules Verne University of Picardie, Amiens, France; ^4^ Department of Cardiology, Center Cardio-Thoracique de Monaco, Monaco, Monaco; ^5^ Cardiac Surgery Department, Centre Hospitalier Régional et Universitaire de Lille, Lille, France

**Keywords:** primary mitral regurgitation, echocardiography, valvular regurgitation, regurgitant volume, regurgitant fraction, heart valve disease (HVD)

## Abstract

The accurate quantification of primary mitral regurgitation (MR) and its consequences on cardiac remodeling is of paramount importance to determine the best timing for surgery in these patients. The recommended echocardiographic grading of primary MR severity relies on an integrated multiparametric approach. It is expected that the large number of echocardiographic parameters collected would offer the possibility to check the measured values regarding their congruence in order to conclude reliably on MR severity. However, the use of multiple parameters to grade MR can result in potential discrepancies between one or more of them. Importantly, many factors beyond MR severity impact the values obtained for these parameters including technical settings, anatomic and hemodynamic considerations, patient's characteristics and echocardiographer' skills. Hence, clinicians involved in valvular diseases should be well aware of the respective strengths and pitfalls of each of MR grading methods by echocardiography. Recent literature highlighted the need for a reappraisal of the severity of primary MR from a hemodynamic perspective. The estimation of MR regurgitation fraction by indirect quantitative methods, whenever possible, should be central when grading the severity of these patients. The assessment of the MR effective regurgitant orifice area by the proximal flow convergence method should be used in a semi-quantitative manner. Furthermore, it is crucial to acknowledge specific clinical situations in MR at risk of misevaluation when grading severity such as late-systolic MR, bi-leaflet prolapse with multiple jets or extensive leak, wall-constrained eccentric jet or in older patients with complex MR mechanism. Finally, it is debatable whether the 4-grades classification of MR severity would be still relevant nowadays, since the indication for mitral valve (MV) surgery is discussed in clinical practice for patients with 3+ and 4+ primary MR based on symptoms, specific markers of adverse outcome and MV repair probability. Primary MR grading should be seen as a continuum integrating both quantification of MR and its consequences, even for patients with presumed “moderate” MR.

## 1. Introduction

The landscape of primary mitral regurgitation (MR) has evolved significantly over the past years (1). First, the global burden of primary MR has increased worldwide: approximately 24.2 million people are affected, with higher absolute prevalence accompanying population aging (2). In high-income countries, primary MR is most commonly caused by myxomatous degeneration due to fibroelastic deficiency or Barlow's disease (3). Conversely, although the prevalence of rheumatic mitral heart disease has decreased, it still remains the main cause of MR in low- and middle-income countries (4). Accordingly, in a contemporary prospective European survey involving 28 countries, the prevalence of primary MR, mainly due to degenerative disease, was found to be approximatively 14% (5).

Surgical correction, preferentially by mitral valve (MV) repair when feasible, remains the sole effective treatment for patients with severe primary MR (6). The preservation techniques of MV repair based on the original principles of MV reconstructive surgery described by Carpentier have improved dramatically in recent years, together with surgeon experience (7). Moreover, video-assisted minimally invasive and robotic tele-manipulation have come forward as less traumatic ways to perform MV surgery (8, 9). Consequently, the contemporary mortality risk of MV repair for primary MR is <1% for the vast majority of patients with primary MR (10). On the other hand, percutaneous transcatheter edge-to-edge repair (TEER) is now established as a validated treatment option in selected patients with a contraindication for surgery (11, 12). At the same time, studies derived from large observational cohorts have underlined major drivers of worse outcome in patients with primary MR due to prolapse, even when asymptomatic (13–15). Hence, according to guidelines issued by both the ESC (European Society of Cardiology) and the AHA (American Heart Association)/ACC (American College of Cardiology), surgery is indicated (class I) for patients with primary MR who are symptomatic and/or present markers of severity such as left ventricular (LV) systolic dysfunction (16, 17). For the AHA/ACC, all patients with severe MR and normal LV function have a class IIa indication for surgery if there is a high probability of MV repair (>95%) and a low operative risk (expected mortality < 1%) (18). In all likelihood, indications for MV surgery can be expected to extend in the years to come.

Hence, accurate assessment of MR severity is today more important than ever in order to determine the best time for surgery in these patients (19). Transthoracic echocardiography (TTE) is the mainstay imaging modality for assessing the mechanism, etiology, severity, and repair probability of MR (20). When TTE is suboptimal or inconclusive for MR quantification, cardiac magnetic resonance imaging (CMR) and/or transesophageal echocardiography (TEE) provide complementary information. Indeed, TEE helps in grading MR severity, although its strongest advantage over TTE or CMR is to offer a comprehensive assessment of the anatomical lesions and mechanism(s) of MR, especially when planning MV surgery (21). The role of exercise TTE is to ascertain key information on the clinical and hemodynamic tolerance of MR rather than to help in quantifying MR severity (22).

The purpose of this work is to provide a practical appraisal of the grading of primary MR by echocardiography. We review the strengths and limitations of the echocardiographic parameters used to assess primary MR in the light of contemporary data. Also we highlight the importance of considering MR from a hemodynamic perspective by the calculation of MR regurgitant fraction (RegFrac), a key parameter of MR severity which has been given renewed attention in recent years (23–26). Finally, we share current views on the latest technical innovations in echocardiographic grading of MR severity, focusing on future avenues of research.

## 2. Echocardiographic workflow for the assessment of primary MR severity

**Figure 1:**
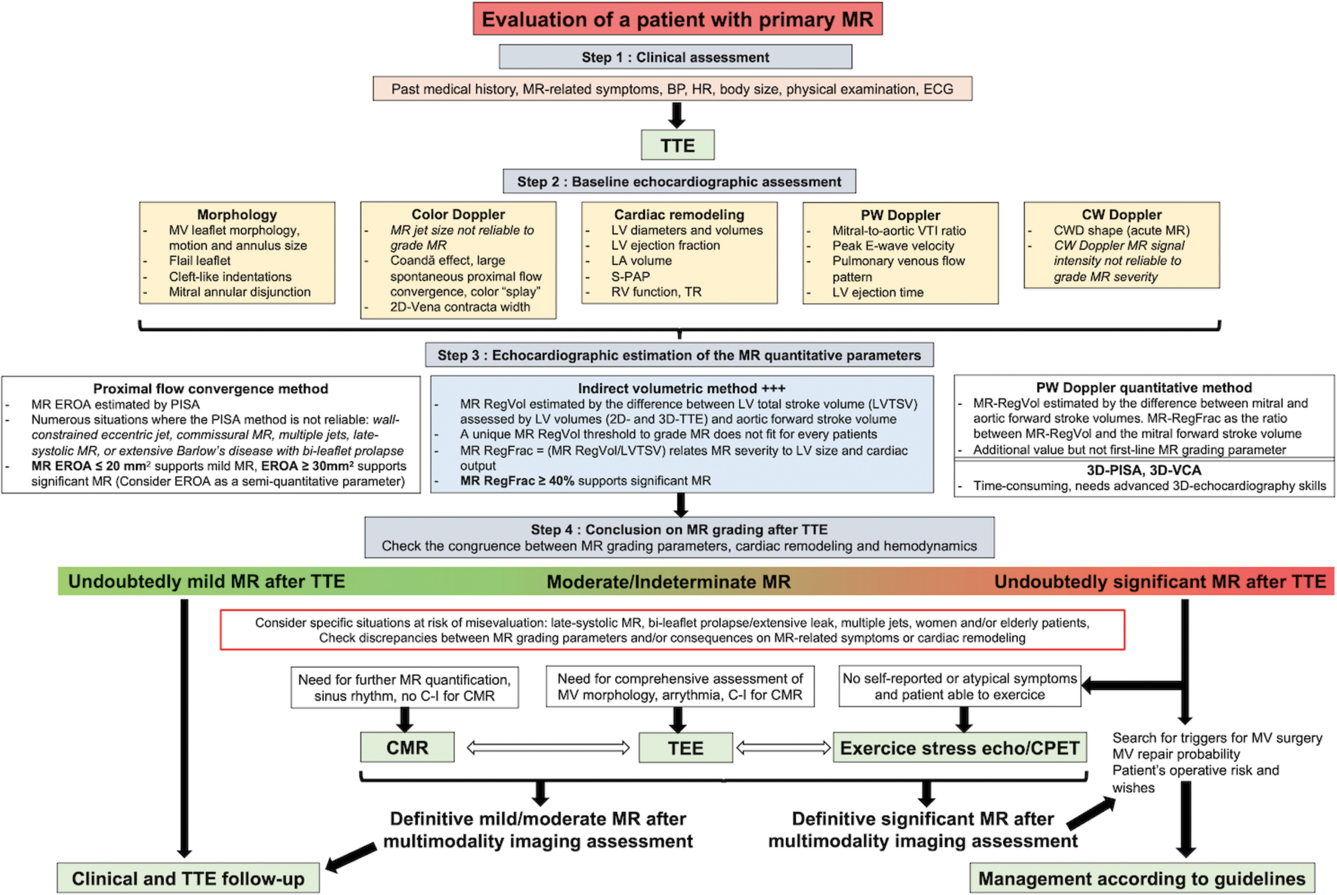
Central algorithm of the echocardiographic assessment of a patient with primary MR. Freely inspired by Zoghbi et al and Hagendorff et al. (23, 28). 2D, two-dimensional; 3D, three-dimensional; BP, blood pressure; CMR, cardiac magnetic resonance; CPET, cardiopulmonary exercice testing; CWD, Continuous-Wave Doppler; ECG, electrocardiogram; EROA, effective regurgitant orifice area; LA, left atrial; LV, left ventricular; MR, mitral regurgitation; MV, mitral valve; PISA, proximal isovelocity surface area; RegFrac, regurgitant fraction; RegVol, regurgitant volume; RV, right ventricular; S-PAP, systolic pulmonary artery pressure; TR, tricuspid regurgitation; TSV, total stroke volume; TTE, transthoracic echocardiography; TEE, transesophageal echocardiography; VCA, vena contracta area; VTI, velocity-time integral.

Both ESC/EACVI (European Association of Cardiovascular Imaging) and ACC/AHA/ASE (American Society of Echocardiography) guidelines prone an integrated multiparametric approach to evaluate MR severity since no single parameter perfectly reflecting MR severity in all patients has been identified (16, 17, 27–31). The echocardiographic parameters used to grade MR are classically divided into three main categories: qualitative, semi-quantitative and quantitative. The qualitative approach consists in the search for “red flags”, that is echocardiographic signs highly specific of severe MR but lacking sensitivity. The semi-quantitative echocardiographic parameters are measurements indicative of MR grade (mild or severe) but including a large intermediate range of values where no conclusion on MR severity can be made. Finally, the quantitative approach intends to estimate the key components of MR which are the mitral effective regurgitant orifice area [EROA], regurgitant volume [RegVol] and regurgitant fraction [RegFrac]. Herein, we provide a practical approach of the grading of primary MR by echocardiography in four steps, derived from our shared clinical experience and in accordance with guidelines (Figure 1). Thorough this entire section, the reader can refer to Tables 1, 2 which summarize the acquisition methods, strengths, weaknesses, and the clinical values of the MR echocardiographic parameters discussed in the following paragraphs.

**Table 1:** Doppler-based echocardiographic parameters used for MR severity grading.

Parameter	Acquisition method	Strengths	Weaknesses
**Color Doppler assessment**			
MR distal jet	– Every apical (4, 2, and 3-chamber) views – Zoom on the MV and LA – Reduce sector as narrow and imaging depth as short as possible to increase framerate – Set color velocity scale as high as possible (high-velocity MR jet) – Set Nyquist limit (aliasing velocity) between 50 and 70 cm/s (default position in the middle of the color bar) – Keep the baseline settings for transducer frequency and pulse repetition frequency (PRF) – Optimize color gain to ensure adequate visualization of MR jet while removing noise from non-moving structures	– Easy and quick to acquire – Allows to detect MR, its origin and jet direction(s) – (Importance of apical 2-chamber and parasternal short-axis views to detect MR commissural jet) – A Coandă effect, a large “spontaneous” (Nyquist limit between 50 and 70 cm/s) proximal flow convergence during the whole systole or color Doppler “splay” suggest significant MR – A clear very small central jet without proximal flow field or visible leaflet abnormalities suggests mild MR.	– MR distal jet not reliable to grade MR – Influenced by flow momentum (MR orifice area × velocity^2^) and driving pressure between LV and LA (check systolic blood pressure), and blood viscosity – Influenced by Doppler color flow technical settings (gain, velocity scale, pulse repetition frequency, transducer frequency, and wall filter) – Underestimates MR severity in case of eccentric or wall-impinging jet (Coandă effect), severe LA dilatation or acute severe MR with high LA pressure – Overestimates MR severity in case of non-holosystolic, multiple jets, or central jet with normal LA pressure (high-velocity jet)
2D-Vena contracta width	– TTE Parasternal long-axis view, biplane measurement on 4- and 3-chamber view (perpendicular to the commissural line), or TEE long-axis view (120°) – Clearly visualize the three components of the MR jet: proximal flow convergence area, vena contracta and jet (angulate the probe out of the standard imaging planes if needed) – Zoom on the MV and surrounding structures – Reduce sector as narrow and imaging depth as short as possible to increase framerate, set color Doppler gain just below the threshold where noise occurs – Set Nyquist limit at the default position in the middle of the color bar (50–70 cm/s) – Measure the narrowest portion of the jet where the largest flow convergence is seen, that is just below the flow convergence area, orthogonal to the direction of the jet. Average at least 3 measures	– A value of 2D-VCW < 3 mm suggests mild MR, >7 mm (8 mm for biplane measurement) suggests significant MR – May be useful in very eccentric wall-constrained jets – Quick to acquire – Feasible even in case of concomitant aortic valve insufficiency	– Wide “grey zone” for intermediate values (between 3 and 7 mm) – Easy to misclassify MR severity with small measurement errors: the VCW should not be measured if the three components of the MR jet are not clearly seen – Moderate reproducibility on TTE (better on TEE) – Limited value in case of atrial fibrillation (average multiple measurements) or mitral calcification – The measurement of 2D-VCW is less reliable when done orthogonal to the direction of the ultrasound beam and distal to the probe because the lateral resolution is lower than the axial resolution in echocardiography – Single-plane measurement of a 3D regurgitant orifice which is not circular-shaped: VC is only a surrogate of the true mitral regurgitant orifice. – Not applicable to multiple jets – Dependent on color Doppler technical settings – Overestimates MR severity in case of non-holosystolic jet
**Continuous-Wave Doppler assessment**			
CW Doppler MR envelope	– Perform multiple acquisition windows to align the ultrasound beam with the MR jet as best as possible – In case of preserved LVEF, peak MR jet velocities by CW Doppler typically range between 4 and 6 m/s: a value of a least 5 m/s should be reached, suspect misalignment of the ultrasound beam otherwise – Use gray scale and set CWD gain as appropriate to optimize signal density without noise artifacts – Use non-imaging high-frequency transducer (Pedof) in right parasternal window in case of posterior leaflet prolapse with anterior-oriented MR jet.	– Easy and quick to acquire (more challenging in very eccentric jets) – Allows to assess timing and duration of MR jet throughout systole (holo-, early- or late-systolic) – Suspect severe MR in case of short and triangular-shaped CWD MR jet (acute severe MR)	– CWD MR signal intensity not reliable to grade MR – Risk of incomplete CW Doppler envelope with under-estimation of peak velocity in case of eccentric jets – Influence of gain on CWD signal density – Influence of systolic blood pressure on peak MR jet velocity
**Pulsed-Wave Doppler assessment**			
Mitral inflow pattern and the mitral-to-aortic velocity-time integral ratio (MAVIR)	– Apical 4-chamber view (5-chamber for LVOT VTI) – Set the sample volume at the tip of the mitral leaflets for mitral VTI (1 cm below the aortic valve for LVOT VTI) – Sweep speed 50 to 100 cm/s – Optimize gain – Acquire VTI_mitral_ and VTI_LVOT_	– Easy and quick to acquire – Highly reproducible – Feasible in eccentric or multiple jets – Excellent diagnostic value to exclude severe MR in case of MAVIR < 1 or in the presence of impaired relaxation pattern (A wave dominance) – High MAVIR > 1.4 suggests significant MR	– Not applicable when atrial fibrillation – Wide “grey zone” for intermediate values of MAVIR (1 to 1.4) – Not applicable to secondary MR, atrial fibrillation, any degree of mitral stenosis or calcification, associated aortic regurgitation – Dependent on loading conditions (LV filling pressure, ejection fraction, LA compliance) – Influence of age on E wave (decreases with aging)
Pulmonary vein flow systolic reversal pattern	– Apical 4-chamber view (angulate the probe to visualize the right upper pulmonary vein on TTE). Try to record on TTE at least one other pulmonary vein (the 4 veins should be checked on TEE) – Set color box on the right upper pulmonary vein – Reduce velocity scale – Set the sample volume of PW Doppler 1 cm deep into the right upper pulmonary vein (TTE). – Set filter gain as appropriate – If the MR jet is visualized toward this vein (usual in posterior prolapse with Coandă), also try to obtain signal from one left pulmonary vein (harder to get on TTE) – Acquire pulmonary venous flow pattern: systolic (S), diastolic (D) and atrial (Ap) waves *(under normal conditions: S and D waves are positive, S > D)*	– Easy to acquire – Feasible in eccentric or multiple jets – Pulmonary vein flow systolic reversal (PVFSR) suggests significant MR [blunting of the pulmonary vein systolic wave (S) is not enough to suggest significant MR] – The 4 pulmonary veins can be analyzed in TEE	– False positive if the MR jet (even in case of moderate MR) is directly oriented towards the sampled pulmonary vein (record other pulmonary veins when possible) – PVFSR pattern largely depends on LA compliance and LV filling pressure: large V waves may occur in the absence of significant MR and vice versa. – PVFSR pattern could be absent despite significant MR in case of severe LA dilatation – Not reliable if atrial fibrillation

**Table 2:** Echocardiographic methods used to estimate MR quantitative parameters.

Parameter	Acquisition method	Strengths	Weaknesses
2D-Proximal Isovelocity Surface Area (=PISA) method	– All available views – Zoom on the flow convergence area – Shift baseline Nyquist limit < 10% of the peak MR jet velocity (typically between 20 and 40 cm/s), towards the direction jet (i.e. downwards for TTE apical views, upwards for parasternal long axis views if posterior prolapse or TEE views) to obtain a clear hemispheric flow convergence area with delimited isovelocity shell *(this is required to satisfy the mathematical assumptions underlying the PISA method)* – *(The 20–40 cm/s range to set aliasing velocity approximatively corresponds to 10% of the peak MR jet velocity. Therefore the aliasing velocity should be optimized within this range according to MR flow velocity. The lower the aliasing velocity, the higher the PISA radius)* (32, 33) – Increase Doppler color smoothing sometimes helps to better delimitate the aliasing isovelocity shell – Use cine-loop frame by frame to select the best-defined PISA – Measure PISA radius from the base of the hemisphere to the point of color aliasing, directly towards the probe (34). (The location of the regurgitant orifice can be hard to detect: it is useful to use simultaneous mode with and without color Doppler to better visualize the MV plane when measuring the PISA radius but it should be kept in mind that the hemodynamic and anatomical regurgitant orifice are often different) – Obtain the MR jet envelope spectrum by CW Doppler. All echocardiographic views should be interrogated to obtain the most complete envelope (it is possible to acquire the CWD MR jet envelope from another view that where the PISA radius has been measured). A value of peak MR jet velocity of at least 5 m/s in case of normal LV ejection fraction should be obtained. The view chosen to acquire PISA radius can be different from the one selected to obtain the most complete CWD MR jet envelope – [*If the CWD MR signal is not available or incomplete, the calculation of MR EROA can be simplified using the following formula: EROA = (PISA radius)^2^/2, assuming a peak MR jet velocity of 5 m/s and an aliasing velocity of 40 cm/s]* (35) – Calculate MR flow rate, mitral EROA and RegVol (see text for formula)	– Outcome data on large epidemiological cohort studies – Independent of MR flow rate and driving pressure – MR EROA ≥ 30 mm^2^ supports significant MR, while MR EROA ≤ 20 mm^2^ supports mild MR – A large PISA radius (≥1 cm) for an aliasing velocity of 30–40 cm/s is highly indicative of severe MR. – Automatic calculations of EROA and RegVol by PISA implemented in echocardiograms – Feasible in case of associated aortic regurgitation – Theoretically feasible in case of atrial fibrillation (however measurements vary from one cardiac cycle to another so average at least 5 consecutive cycles)	– MR EROA by PISA should be considered as a semi-quantitative parameter (proportional relationship between increase in EROA or RegVol at an epidemiological scale but significant variability at an individual level) – MR RegVol by PISA should not be reported – Several specific features in primary MR where the PISA method is not reliable (see text) – The PISA method relies on idealized conditions which are rarely encountered *in vivo*: the shape of the regurgitant orifice is very often non-hemispheric but rather hemi-elliptic, “urchinoid”, to even more complex curvilinear (“smiley-like”) anatomy, therefore all measurements by PISA are only approximations (36, 37). – The shape of the isovelocity shell depends on the aliasing velocity: a velocity set too low or too high results in an elongated or flat shell, respectively (38) – The value of PISA radius varies according to the selected frame to measure it (mid-systolic versus the one where the largest radius is visualized) (38). – The definition of the base of the flow convergence from which the radius is measured varies among studies (either the vena contracta, the anatomical level of the regurgitant orifice or the color base of the flow convergence) (38). – PISA radius is squared in the formula used to estimate MR flow rate, therefore even small error measurements (1 or 2 mm) can lead to high magnitude errors – Dropout at the angles of the flow convergence due to underestimated velocities (“Doppler angle effect”) (40). – Not reliable in case of multiple jets (it is theoretically possible to add PISA radii of separate jets, but this has not been clinically validated) (39). – The flow convergence can be constrained by adjacent structures such as the LV wall (notably in posterior valve prolapse with eccentric jet or commissural MR) or annular calcification, thereby resulting in a “oblong” shape (angle correction methods have been suggested but in practice there is no consensus on how the angle should be measured) (39, 41, 42). – The calculation of mitral EROA and RegVol are based on a single time point measurement (PISA radius) therefore not considering the dynamic variations of MR throughout systole. – Influence of Doppler angulation on isovelocity surface areas (risk of under-estimation if there is an angle between the line of the ultrasound beam and the flow).
2D-TTE volumetric method	– Apical 4-chamber and 2-chamber views for LV volumes according to the biplane Simpson's method – Adjust settings to optimize as best as possible LV endocardial definition (ultrasound contrast could help when poor echogenicity), avoid apical foreshortening – Trace LV borders in end-diastole and end-systole at the interface between the LV cavity and the compacted myocardium (not at the blood-tissue interface). Estimate LV total stroke volume – Parasternal long axis and Apical-5-chamber views for forward aortic stroke volume measurement – Zoom on aortic valve, open leaflets, inner edge-to-inner edge, measure LVOT diameter (*Δ*_LVOT_) at mid-systole – Set the PW Doppler sample volume 1 cm below the aortic valve for aortic VTI [VTI_LVOT]_) – Calculate aortic forward systolic stroke volume (ml) = π x (ΔLVOT)² x VTILVOT/4 – Calculate MR RegVol and RegFrac	– Important clinical value: the determination of RegFrac allows to appraise MR severity from a hemodynamic perspective – Indirect method, thereby feasible even in case of very eccentric or multiple jets – Independent from dynamic variations of MR during systole – Finding different RegVol values estimated by 2D-volumetric versus PISA methods is not surprising – Serial assessment of MR severity using volumetric methods (comparison of mitral RegVol and RegFrac values for a given patient over time) is possible under strict methodological conditions	– Learning curve to acquire 2D-TTE LV volumes values close to CMR ones (similar LV stroke volume between 2D-Simpson's BP and forward aortic stroke volume in normal patients) – Lack of reliable echocardiographic apical windows in some patients – Less reliable in case of atrial fibrillation – MR-RegFrac 40% threshold highly specific of significant MR, but large grey zone of overlap to discriminate 3 + 4+ MR from 1 + 2+ MR – Depends upon LV geometry assumptions – Relies on the assumption of a circular shape for LVOT when estimating LVOT area (the true shape of LVOT is rather elliptical) – Risk of imprecision when measuring LVOT diameter, particularly in case of associated aortic calcification – Presence of a “prolapse volume” in bi-leaflet prolapse with mitral annulus disjunction that may result in some degree of measurement bias – Higher inter- and intra-observer variability than CMR measurements – Limited outcome data for TTE volumetric method (available for CMR)
3D-TTE volumetric method	– Apical 4-chamber view. A good image quality in 2D is required before performing 3D mode – Adjust the size of the sector to cover the entire LV cavity and myocardium – Perform semi- or automated 3D-quantification of the LV endocardial borders – Check whether the tracking of LV endocardium is adequate throughout the cardiac cycle with correct LV end-diastolic and end-systolic volumes. Estimate LV total stroke volume – Estimate aortic forward stroke volume (see 2D-TTE volumetric method) – Calculate MR RegVol and RegFrac	– Indirect method, thereby feasible even in case of very eccentric or multiple jets – Independent from dynamic variations of MR during systole – Closer RegVol values from those obtained with CMR quantitative method compared with 2D-TTE volumetric methods – It is also possible to estimate the mitral RegVol as the difference between LV and RV stroke volumes (if there is no other left or right valve regurgitation). However, the accurate assessment of RV volumes by 3D-TTE can be very challenging or even not feasible in routine practice.	– Decreased temporal (if multibeat acquisition not achieved) and spatial resolution compared with 2D – Possible stitching artifacts (incomplete hold-breathing, motions of the patient or the probe, arrhythmia) – Better reproducibility with LV volumes acquired with 3D- versus 2D-TTE volumetric methods – Reduced grey zone of overlap for the discrimination of 1 + 2+ MR from 3 + 4+ MR compared to 2D-TTE volumetric method – Inter-vendor discrepancies in the assessment of LV volumes by 3D-TTE. – 3D-TTE can be limited by insufficient acoustic window – Not applicable in case of atrial fibrillation – Same concerns than 2D-TTE about LVOT diameter measurement
Pulsed-Wave Doppler quantitative method	– Apical 4-chamber view (5-chamber for LVOT VTI) – Measure LVOT diameter (*Δ*_LVOT_) as mentioned above (2D-TTE volumetric methods), measure mitral annulus diameter (*Δ*_MV_) at early-diastole – Set the PW Doppler sample volume at the level of the mitral annulus for mitral VTI [VTMV_]_ [1 cm below the aortic valve for LVOT VTI (VTI_LVOT)_] – Acquire mitral (VTI_mitral)_ then LVOT VTI (VTI_LVOT_) (gain should be optimized because the quality of the PW Doppler spectral envelope and manual tracing impact the aortic and mitral VTI values; sweep speed 50 to 100 cm/s) – Calculate LV total stroke volume (ml) = π x (ΔMV)² x VTIMV/4 – Calculate aortic forward systolic stroke volume (ml) = π x (ΔLVOT)² x VTILVOT/4 – Calculate MR RegVol and RegFrac	– “Historical” quantitative approach used as comparative method for qualitative and semi-quantitative MR indices in landmark studies – Indirect method, thereby feasible even when very eccentric or multiple jets – Independent from dynamic variations of MR during systole – Outcome data – Useful as an additional parameter in case of severe inconsistencies between other approaches	– Not recommended as first quantitative approach because it is time-consuming and with numerous sources of error measurements: the calculation of the transmitral volume roughly estimates the true amount of blood flowing across the MV. – Only in sinus rythm – Not feasible in case of associated aortic regurgitation [*it is theorically possible to determine effective stroke volume by the PW Doppler spectral envelope of the right ventricular outflow tract (RVOT) VTI and the measurement of RVOT diameter.]* (43) – Both the mitral and aortic annulus are assumed of circular shape (44). – The shape of the mitral annulus varies throughout systole and with an apex-to-base motion (45, 46). – The measurement of the mitral annulus in the apical-4-chamber view could suffer from significant interobserver variability according to the orientation of the ultrasound probe.

### 2.1. Clinical assessment

The evaluation of a patient with MR begins with a full report of her/his past medical history, previous medication regimens and a complete physical examination with heart auscultation and electrocardiography (ECG). Blood pressure, heart rate and rhythm must be recorded before echocardiography. Search for potential valve-related symptoms such as dyspnea requires experience, especially in older patients who are likely to limit themselves in daily activities without reporting any complaint at the time of evaluation. Standardized questionnaires can be helpful (47).

### 2.2. Baseline echocardiographic assessment

#### 2.2.1. Morphological assessment

Before to compute any measurement, the full assessment of all components of the MV apparatus (leaflets, annulus, sub-valvular apparatus) is critical. The mechanism(s) underpinning mitral loss of coaptation are evaluated according to Carpentier's classification of leaflet motion. This approach allows to distinguish patients with primary MR (due to organic leaflet abnormalities) to those with secondary MR (due to LV dysfunction and/or high LA pressure) (20). It is relatively easy to classify MR as primary in in the presence of obvious valve abnormalities such as prolapse, perforation, flail, or papillary muscle rupture. In patients with Barlow's disease, the presence of a mitral annulus disjunction should be carefully checked notably because of its implications for arrhythmic risk stratification (48, 49). It may be more difficult to elucidate whether MR is “primary” or “secondary” in the presence of single- or bi-leaflet retraction which is usually associated with a dilated mitral annulus (50). Indeed an initially secondary MR can worsen leading to so-called “tertiary” or “mixed” MR (51). Nowadays, 3D-echocardiography imaging provides the best morphologic information for the MV complex, as comprehensively discussed elsewhere (52).

##### 2.2.1.1. Flail leaflet

**Figure 2:**
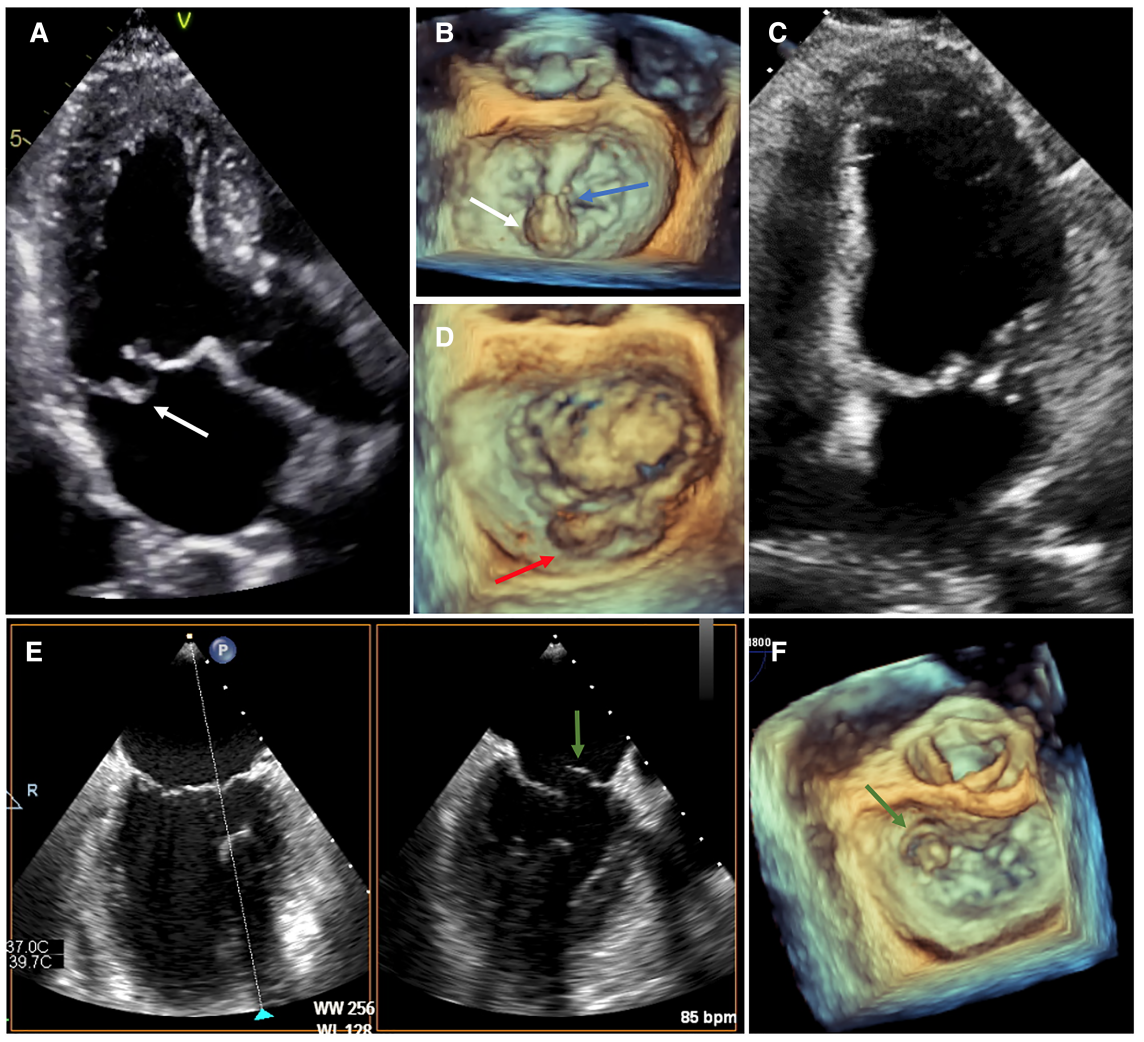
Morphological assessment of flail leaflet. (**A,B**) Patient with posterior flail leaflet visible on TTE (the tip is directed towards the LA). TEE confirms the involvement of segment P2 (white arrow) with chordae rupture (blue arrow). (**C,D**) Patient with bi-leaflet MV prolapse and P2 flail (red arrow) only visible on 3D-imaging TEE (surgical “en face” view). (**E,F**) Patient with flail involving the segment A1 (green arrow) visible on TEE imaging using biplane mode from the bi-commissural view (60–90°), and on 3D-imaging (surgical “en face” view). LA, left atrium; TTE, transthoracic echocardiography; TEE, transesophageal echocardiography.

The presence of a flail leaflet is highly supportive of severe MR and associated with worse outcome (53–55). Also flail was the key inclusion criterion in the original MIDA cohort (56). Whether all patients with flail have severe MR has been recently a source of debate (57, 58). Importantly, the morphological features of chordae rupture and flail in MR should be considered (59). Indeed, sometimes small chordae may rupture and lead only to moderate MR. Sometimes, big chordal rupture occurs, but because of the redundancy and hypermobility of the opposite leaflet the coaptation gap is not big and MR is only moderate. Hence, the accepted echocardiographic signature of flail leaflet requires not only chordae rupture but also clear visualization of a rapid systolic movement of the involved leaflet tip towards the left atrium (LA) (Figure 2). In practice, flail leaflet can be over-detected by TTE when the prolapsed leaflet is not entirely seen, such that the observed distal part of the leaflet seems to be directed towards the LA whereas the real tip looks towards the LV. Further research using advanced 3D-imaging techniques would be of great interest to better appreciate the relationship between the anatomic features of flail leaflet and MR severity.

##### 2.2.1.2. Cleft-like indentations

Cleft-like indentations (CLI) are deep separations extending ≥50% of the mitral valve leaflet depth in myxomatous valve prolapse (60). They are difficult to detect on standard echocardiography and are best assessed using 3D-imaging (52, 61, 62). CLI are important to treat during surgery otherwise they may cause residual MR following intervention.

##### 2.2.1.3. Papillary muscle rupture

Acute severe MR secondary to papillary muscle rupture is a rare but deadly condition which requires urgent surgery (63). Although it is most frequently due to acute myocardial infraction (AMI), some cases of spontaneous papillary muscle rupture have been reported (64, 65).

*Main message for the clinician*

*“*First look at the valve !” when evaluating a patient with MR. Noteworthy, the distinction between primary and secondary MR is not always straightforward. In particular, the progressive aging of patients referred for evaluation can result in more complex degenerative MR presentations not limited to prolapse or flail but also involving extensive calcifications of the mitral annulus, possible reduced echogenicity because of an insufficient acoustic window, restricted mobility, or even some degree of associated mitral stenosis (66). Echocardiographic assessment of MR severity in these patients can be quite challenging.

#### 2.2.2. Color Doppler assessment

##### 2.2.2.1. MR distal jet

**Figure 3:**
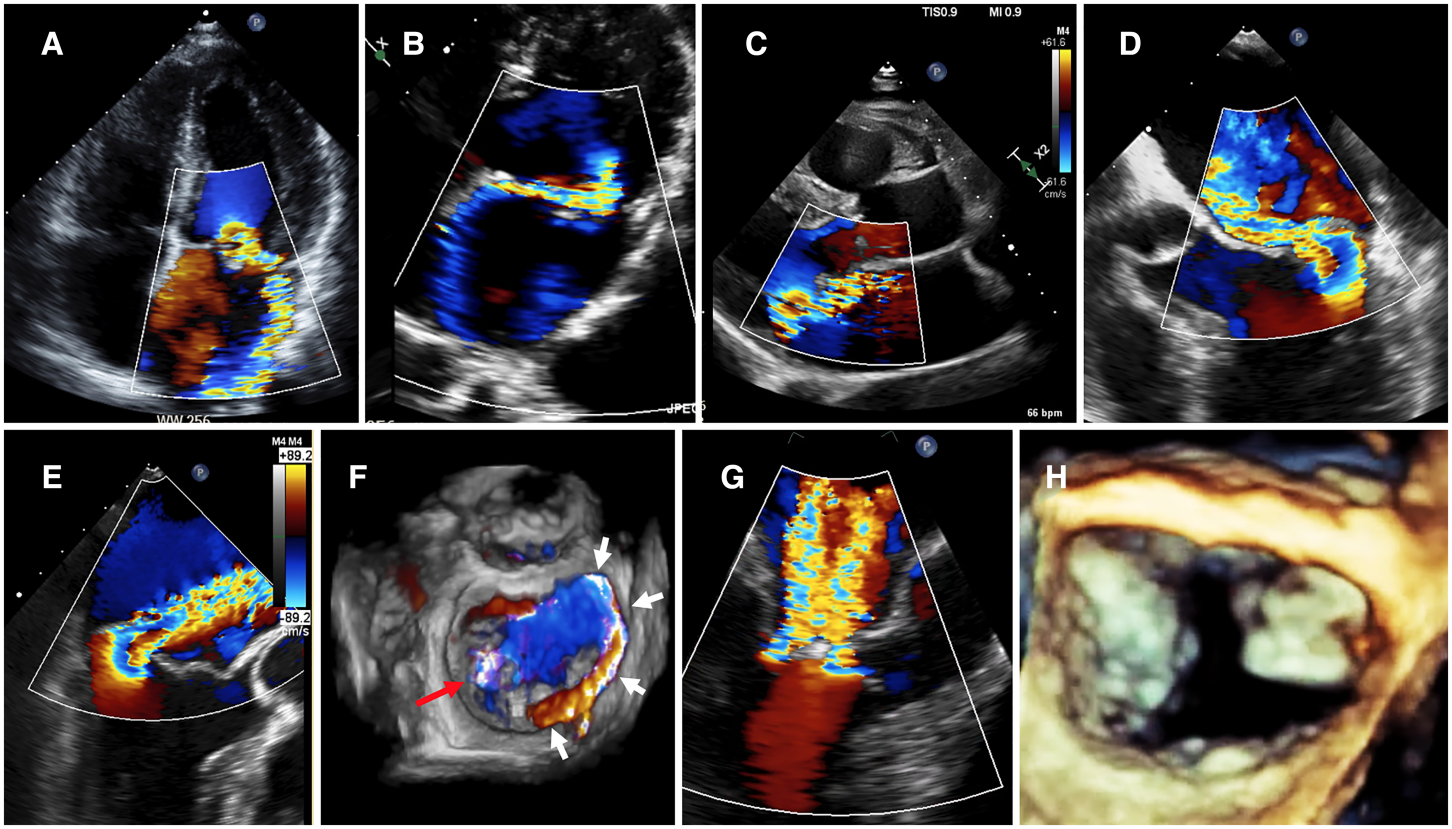
Doppler color approach of primary MR evaluation. (**A**) Anterior MV prolapse with eccentric MR jet impinging on the posterior LA wall because of Coandă effect. The direction of the MR jet helps in understanding the prolapse's mechanism. (**B**) Posterior MV prolapse with eccentric jet impinging on the interatrial septum down to the pulmonary veins. Despite a small color flow jet area, a significant MR should be suspected because of the presence of a Coandă effect. (**C**) Posterior MV prolapse with large spontaneous proximal flow convergence (i.e without modifying Doppler color settings). A significant MR should be suspected. (**D,E**) Examples of posterior MV flail by TEE where the 3 components of the MR are clearly visualized (proximal flow convergence, vena contracta and distal MR jet). Hence, it is possible to measure 2D-vena contracta width. However, it should be kept in mind that the measurement of 2D-VCW could be less reliable when performed orthogonal to the direction of the ultrasound beam because of lower lateral than axial resolution. (**F**) Surgical “en face” view with Doppler color mode. A full MV 3D color-coded data set can objectively document MR regurgitant(s) jet(s). However, this method could suffer from markedly decreased framerate, therefore multi-beat acquisition is usually required to obtain an acceptable quality imaging. Herein, the origin of the MR is visualized at the MV anterior commissure (red arrow), and the distal MR jet is impinging on the inter-atrial septal wall because of the Coandă effect. (**G,H**) Double MR jet with a pattern of “Crossed swords sign”. 3D-TEE imaging revealed a large anterior cleft indentation. 3D, three-dimensional; LA, left atrium; MR, mitral regurgitation; MV, mitral valve; TEE, transesophageal echocardiography; TTE, transthoracic echocardiography; VCW, vena contracta width.

The use of Color Doppler mode allows to appraise the presence, origin, timing (with M-mode), and direction of the MR regurgitant(s) jet(s). All echocardiographic views should be checked. It is worth using biplane mode from a bi-commissural view (TTE: apical 2-chamber, parasternal short-axis views; TEE: 60–90°) and sweep the Doppler line from one commissure to another across the MV coaptation line with simultaneous Doppler color mode comparison (67). However, only a full MV 3D color-coded data set with sufficient framerate can objectively document MR regurgitant(s) jet(s), especially when several jets (“multi-jet”) are present (68). Color Doppler analysis of the jet(s) helps in understanding the MR mechanism(s) (69, 70) (Figure 3). Indeed an isolated posterior MV prolapse is expected to induce an eccentric anterior MR jet towards the interatrial septum (71). More complex MR mechanism(s) or associated lesions such as cleft-like indentations or commissural leaks should be suspected in case of “atypical” MR direction and/or multiple jets (72–74). Primary MR due to non-P2 prolapse and/or horizontal MR jet are likely to be underestimated by TTE (75).

Although the assessment of the MR jet size (itself or related to LA size) is still very often used as a first method to grade MR severity, this is not a recommended approach (76). Indeed the appearance of MR distal jet on Color Doppler mode strongly depends on technical settings, hemodynamic and anatomical factors beyond MR severity (77–79). Doppler color mode does not image MR flow but rather the spatial distribution of velocity estimates within the plane. Therefore MR severity assessment based only on color Doppler mode of MR distal jet is not recommended.

##### 2.2.2.2. Color Doppler features suggestive of significant MR

On the other hand, several features of MR assessed by Color Doppler mode are suggestive of significant MR. Independently from jet size, the presence of a Coandă effect (eccentric jet impinging on the LA wall towards the pulmonary veins) suggests significant MR but is not pathognomonic (58, 80, 81). A large “spontaneous” (i.e., not changing Doppler color settings) proximal flow field during the whole systole supports significant MR. Recently, a new sign named “color Doppler splay”, consisting in an artifactual horizontal extension of the color Doppler signal, has been shown to be associated with concealed and significant MR (82).

##### 2.2.2.3. 2D-vena contracta width

The 2D-Vena contracta width (2D-VCW) is a single-plane measurement of the narrowest portion of the MR jet at its origin, thereby acting as a surrogate of the true mitral regurgitant orifice (83–86). The clear visualization of the three components of the MR jet (proximal flow convergence area, vena contracta and distal jet) is required to measure 2D-VCW (87). Because in primary MR eccentric jet formations are often present, the severity of MR cannot be quantified by 2D-VCW in most cases. Indeed, when the VC width is measured orthogonal to the direction of the ultrasound beam and distal to the probe, this is less reliable due notably to the issues of lower lateral than axial resolution in echocardiography. In contrast, the measurement of the 2D-VCW could be more reliable when performed along the direction of the ultrasound beam. Also the assumed independency of 2D-VCW from LV-LA driving pressure or regurgitant flow rate for a fixed orifice is questionable (88). Noteworthy the 2D-VCW does not consider the dynamic variations of MR throughout systole because it is measured in a single frame (89). Despite these important limitations, measuring 2D-VCW sometimes helps in grading MR, notably when the PISA method is not reliable because of a wall-constrained flow field.

#### 2.2.3. Continuous-Wave Doppler assessment

**Figure 4:**
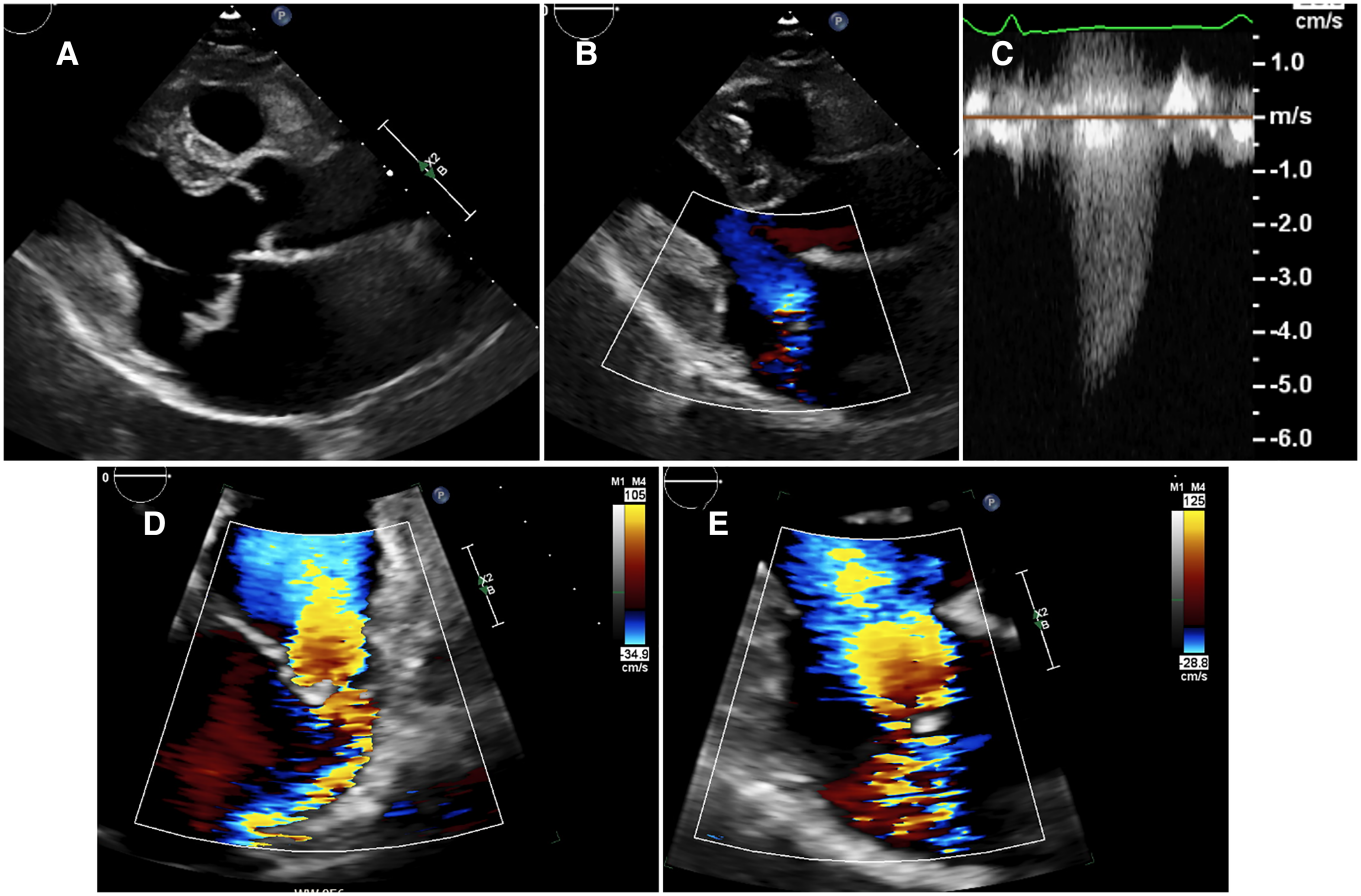
Patient with primary MR with typical features of Barlow's disease. Presence of a MV valve prolapse (A3P3) with mitral annulus dilatation and disjunction (**A**). The MR jet is very eccentric with small color jet size (**B**). The best CWD MR envelope is obtained in the long-axis parasternal view, revealing a mid-late systolic MR (**C**). Using the PISA method, the proximal flow convergence area displays an oblong shape in apical-4-chamber view because being constrained by the LV lateral wall (**D**). The proximal flow field is better delineated in long-axis parasternal view (**E**), with an “urchinoid” shape because of loss of Doppler signal on its angles. CWD, Continuous-Wave Doppler; LV, left ventricular; MR, mitral regurgitation; MV, mitral valve; PISA, proximal isovelocity surface area.

The Continuous-Wave Doppler (CWD) signal of the MR jet is obtained by aligning the Doppler line through the vena contracta, parallel to the jet, with the focus of the CWD at its origin. It is important to record the most complete CWD envelope as possible. However, this can be very challenging in eccentric jets. Indeed in patients with posterior prolapse, the highest envelope density is usually obtained by aligning the Doppler line either in the parasternal long-axis view (CWD ascending signal) or in the LV outflow tract in the apical-5-chamber view. Similarly, an incomplete envelope confined to the latter half of systole could suggest late-systolic MR (Figure 4), while a complete spectrum obtained from another acoustic window would result in reclassification of the MR as holo-systolic. A triangular-shaped CWD MR signal with early peaking and low velocity supports severe MR (90). It can be observed notably in acute severe MR when LA compliance is quickly overwhelmed by excess volume load, resulting in high LA pressure and decreased LV-LA driving pressure. Theorically, the CWD envelope density is proportional to the number of red blood cells passing through the MV, so a dense signal would suggest significant MR (91). However, the CWD signal is affected markedly by settings such as gain, filter or insonation angle. Therefore CWD intensity is not recommended for grading of MR severity (29).

#### 2.2.4. Pulsed-Wave doppler assessment

##### 2.2.4.1. MAVIR

**Figure 5:**
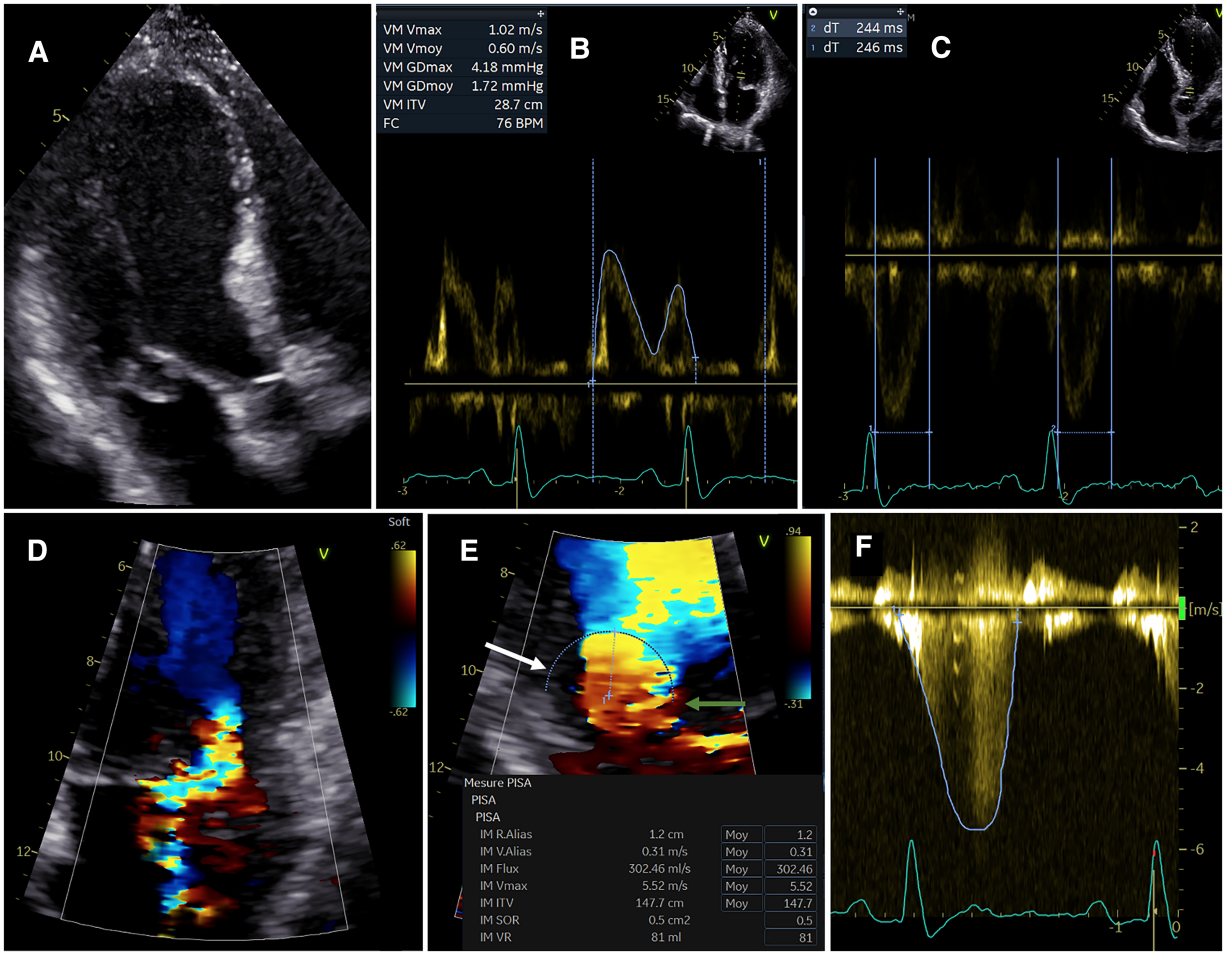
Echocardiographic assessment of MR severity grading. Illustrative example of a 50-year-old male patient with primary MR. Posterior MV flail leaflet is seen on apical-3-chamber view (**A**). The mitral VTI is measured at 28.7 (**B**: the sample volume is positioned at the tip of the mitral leaflets). The LVOT VTI is measured at 17.7 (see Figure 7), thus resulting in a high MAVIR (1.62). A short LV ejection time is observed (<260 ms, **C**). The 3 components of the MR jet (proximal flow convergence, vena contracta and distal jet) are not clearly identified (**D**), therefore the 2D-VCW cannot be measured accurately. The PISA radius is measured at 1.2 cm for an aliasing velocity of 31 cm/s, thus suggesting significant MR. The MR EROA and MR RegVol estimated by the PISA method are of 0.51 cm2 and 81 ml, respectively. (**E**) However, the proximal flow convergence is partly constrained by the posterior LV wall (white arrow). Also there is a dropout of MR signal on the other side of the hemisphere (green arrow) because of a “Doppler angle effect”. Then, the MR is mainly mid-late systolic as shown by the CW Doppler MR signal (**F**). Hence, the MR RegVol by the PISA method is likely to over-estimate the true MR RegVol. All taken together, the MR is likely to be significant. CWD, Continuous-Wave Doppler; MAVIR, mitral-to-aortic velocity-time integral ratio; MV, mitral valve; VTI, velocity-time integral; TEE, transesophageal echocardiography; TTE, transthoracic echocardiography.

The mitral-to-aortic velocity time-integral ratio (MAVIR) is easy, quick to acquire and very reproducible (92). It is the dimensionless ratio of mitral to aortic VTI or in other words a “simplified” approach of the PW Doppler quantitative method (detailed hereafter) because not considering either mitral or LV outflow tract (LVOT) annulus size and shape (93). However, the MAVIR cannot be used in patients in arrythmia, associated aortic regurgitation, and/or with higher mitral inflow velocities which are not attributable to MR, such as in presence of any degree of MV stenosis or annular calcification. Importantly, the PW Doppler sample volume must be positioned at the level of the leaflet tips (not at the mitral annulus, Figure 5). A value of MAVIR > 1.4 suggests significant MR, while a dominant A-wave mitral inflow pattern or a MAVIR < 1 highly supports non-severe MR.

##### 2.2.4.2. Peak E-wave velocity

The peak E-wave velocity positively correlates with MR severity, but its true diagnostic value is questionable (94, 95). Indeed many patients have significant MR despite not reaching the recommended thresholds for E-wave velocity (ASE: 1.2 m/s, EACVI: 1.5 m/s) (96). Notably the normal E-wave velocity decreases with age and is higher in women (97). Conversely, some young individuals without MV disease or patients with mitral stenosis, annular calcification or high LA pressure without MR have E-wave velocities higher than 1.2 m/s. Hence the peak E-wave velocity reflects not only MR severity but also the LV-LA pressure gradient.

##### 2.2.4.3. Shape of the aortic velocity-time integral

The shape and duration of aortic velocity-time integral also should be looked. A small, early-systolic and triangular-shaped aortic VTI suggests significant MR (Figure 5). A short LV ejection time (<260 ms) may identify among patients with moderate or severe MR those at higher risk of MV surgery during follow-up (98).

##### 2.2.4.4. Pulmonary venous flow pattern

The pulmonary venous flow pattern assessed by PW Doppler reflects the pulmonary vein-LA driving pressure, which can be impacted by the presence of significant MR but also by other causes of altered loading conditions or LA volume (99). Thus a complete pulmonary vein flow systolic reversal (PVFSR) pattern and not only blunting should be supportive of significant MR. PVFSR can be confounded with the MR jet if both are along the same line. Also an eccentric or high velocity jet of an only moderate MR may selectively enter a given pulmonary vein, resulting in a false-positive PVFSR. The pulmonary venous flow recording by TEE allows to individually interrogate each of the four pulmonary veins. Itakura et al. recently reported a positive correlation between the number of pulmonary veins where a PVSFR pattern was found, pulmonary capillary wedge pressure, and 3D-vena contracta area (100).

#### 2.2.5. Consequences of MR on cardiac remodeling

A very important parameter in valvular heart disease is the evaluation of the consequences on cardiac remodeling. In practice, a whole standard TTE examination is usually performed before to specifically focus on MR quantitative measurements such as EROA or RegVol. Chronic primary MR causes direct volume overload on the LA and in later stages pressure overload. In addition, the left ventricular stroke volume (LVSV) must compensate for the mitral RegVol to maintain LV forward stroke volume. This will progressively lead to LV dilatation according to the Frank-Starling law. Therefore LV volumes and diameters must be carefully assessed. The evaluation of LV volumes would intuitively better reflect MR consequences on remodeling than diameters. Moreover, patients with primary MR can display spherical mid-to-apical LV end-systolic remodeling that contributes to higher LV end-systolic volume (ESV) despite normal LV end-systolic diameter (ESD) (101). However, LVESD remains a crucial parameter of LV impairment in primary MR that integrates LV dilatation, systolic function and afterload. From an epidemiological perspective, the strong prognostic value of LVESD on outcome has been demonstrated in large scale cohort studies. From a practical perspective, LV diameters assessed in parasternal long-axis view, whenever possible using M-mode, are more reproducible from a center to another than volumes in 2D-TTE. It is important to position the probe as high as possible on the patient's chest (only the LV base should be seen) so as to get the ultrasound beam strictly orthogonal to the LV walls, at the tip of the MV leaflets. Also the assessment of LA volume, a strong predictor of outcome in primary MR, requires great consideration. Especially, standard apical views maximize the long axis of the LV, rather than the dimensions of the atria, resulting in LA foreshortening (102). Therefore, LA-focused apical views should be systematically acquired to provide more reliable estimation of size.

In more advanced stage of the disease (or in case of acute worsening, for example due to sudden chordae rupture), symptoms occur, alongside with LV systolic dysfunction and/or pulmonary hypertension (103). A particular problem of MR is that LV afterload is reduced, so the LVEF remains normal or supranormal until the disease reaches an advanced stage. Therefore, parameters such as LVEF or global longitudinal strain over-estimate LV contractility (104). Also the presence of mitral annulus disjunction may simulate stronger LVEF (105). Presence of a small aortic VTI despite a hyperkinetic LV (LVEF > 60%) owing to low impedance is suggestive of severe MR. A significant primary MR with LVEF below 60% indicates early LV dysfunction and requires prompt surgery. It is difficult to estimate accurately LV filling pressure by echocardiography in primary MR (106). Indeed, E/A and E/Ea ratios are not reliable predictors of LV filling pressure in MR (107, 108). In contrast, the difference between the duration of pulmonary vein and mitral A waves may estimate LV end-diastolic pressure independently from MR (108). Recent studies have suggested that a new staging classification for cardiac damage (also including right ventricle damage) in patients with asymptomatic moderate or severe primary MR would provide independent and incremental prognostic value (109, 110). Strikingly, a recent study derived from the MIDA-Quantitative registry reported that LA dilatation, atrial fibrillation, high pulmonary pressure and/or at least moderate tricuspid regurgitation could be independently associated with post-operative survival in degenerative MR (111). Nevertheless, all these consequences are not specific to chronic severe MR and may result from other cardiac conditions that must be carefully ruled out.

The common belief that chronic primary MR cannot be severe if the LV is not dilated is probably accurate for men, but not necessarily for women and/or elderly patients. Indeed, normal LV volumes decrease with age and are lower in women (112). It is not uncommon in daily practice to observe a normal or only mildly enlarged LV in women or elderly patients despite true chronic symptomatic severe MR. Mantovani et al. reported that normalizing for body size, LV and LA diameters were at least as large in women as in men (113). A recent Asian network analysis of cardiac remodeling in 850 patients with at least moderate chronic MR highlighted a phenogroup of old patients with relatively preserved LV size (114). Further studies focusing on volume data are needed to define the sex- and age-normalized cut-off levels for LV and LA dilatation appropriate for valid prognosis assessment of primary MR.

*Main message for the clinician*

The appraisal of the consequences of MR on cardiac remodeling is as important as the collection of MR specific echocardiographic parameters. A patient with a MR classified as moderate or indeterminate solely on the basis of the TTE findings but with LA/LV enlargement and/or elevated systolic artery pulmonary pressure requires a more thorough assessment. Patient's age and sex should be considered when assessing left heart volumes.

### 2.3. Echocardiographic estimation of the MR quantitative parameters

The quantitative methods as stated by guidelines refer to those allowing to evaluate the three key components of MR severity: (1) mitral effective regurgitant orifice area (EROA) that directly evaluates the area of leaflet coaptation gap; (2) mitral RegVol; and (3) mitral RegFrac, related to the amount of load and the hemodynamic consequences of MR. Despite that the outcome implications of MR quantitative methods have been demonstrated in epidemiological studies, they still remain widely under-used as shown in two surveys (77, 115, 116). Yet as recently reported, quantitative methods are feasible for use in daily practice in MR (117, 118). In the Mayo Clinic cohort, MR quantification either by PISA and/or PW Doppler quantitative methods was feasible in more than 4 patients out of 5 with moderate or severe MR (117). In practice, there are clinical situations where formal quantification is not required to grade MR as “severe”, such as a symptomatic patient with MV flail leaflet and huge coaptation gap. Apart from these specific situations and in accordance with guidelines, we advocate the use of quantitative methods whenever possible when grading MR severity.

#### 2.3.1. Proximal flow convergence method

##### 2.3.1.1. Pathophysiological basis

The proximal isovelocity surface area (PISA) method is based upon the fact that the amount of regurgitant flow through a given orifice can be determined by basic principles of fluid dynamics alongside the aliasing phenomenon (119–121). Indeed when crossing a finite rounded orifice, the blood accelerates in concentric shells with progressive decreasing surface area and increasing velocity. Therefore, the flow through the regurgitant orifice equals the flow through a given isovelocity shell according to the law of conservation of mass. The isovelocity shells can be revealed by modifying the Nyquist limits of the color Doppler settings, which will result in aliasing at the set levels. This allows to visually identify the hemispheric isovelocity shells close to the orifice provided that the aliasing velocity chosen is approximatively 10% of the peak MR jet velocity (that is in practice between 20 and 40 cm/s depending on low- vs. high-flow status) (32). The MR flow rate is estimated by the following formula: 2π × *r*^2^ × *V_r_* where *r* is the PISA radius and *V_r_* the aliasing velocity. By applying the continuity equation one can estimate EROA as MR flow rate/Vmax and RegVol as EROA x VTI where Vmax and VTI are respectively the maximum Doppler velocity and velocity-time integral of the regurgitant jet estimated by CWD mode.

##### 2.3.1.2. Limitations of the PISA method

**Figure 6:**
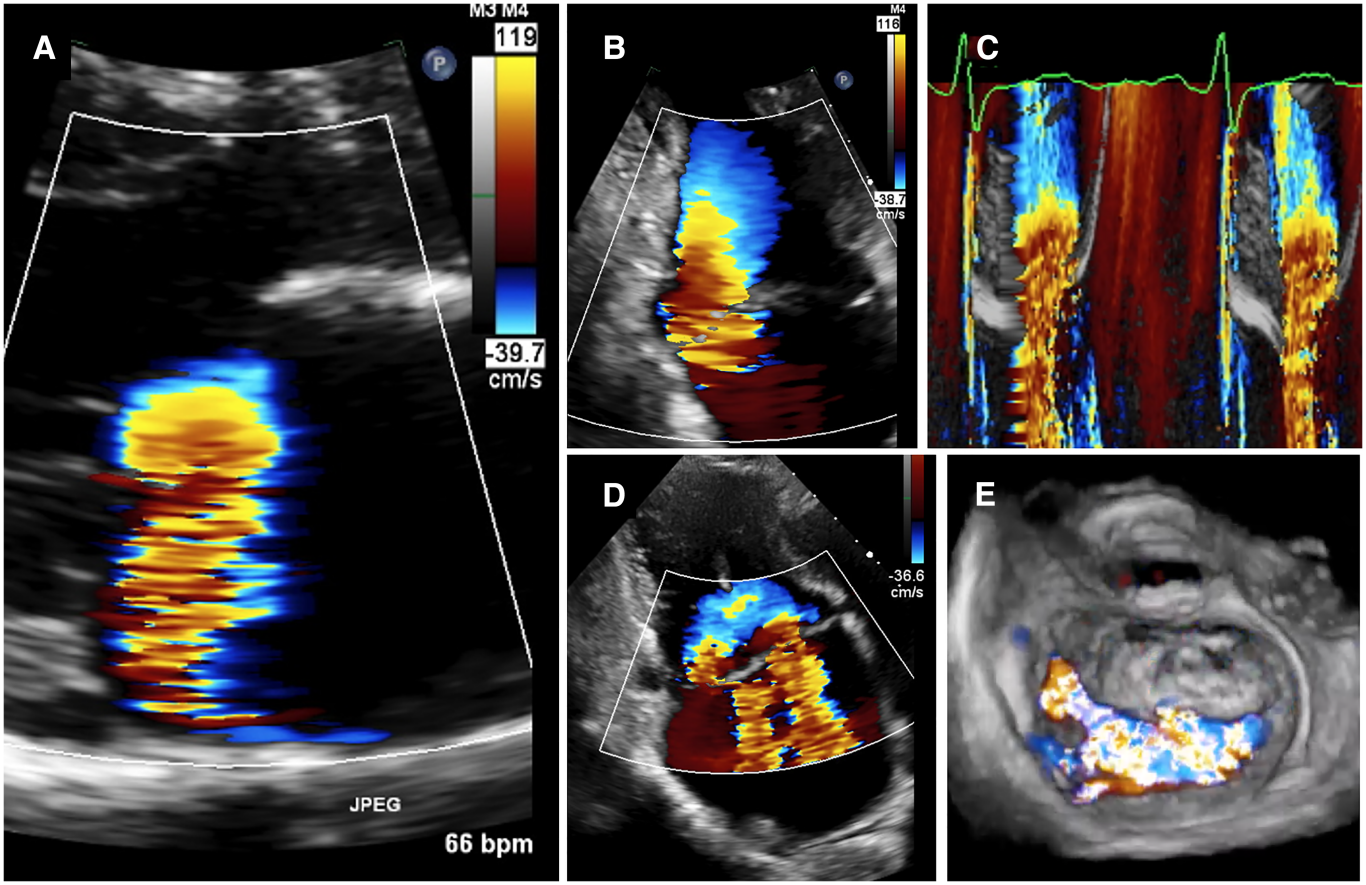
Examples of pitfalls of the PISA method. (**A**) Patient with anterior MV prolapse. A clear hemispheric-shaped proximal flow convergence is seen on parasternal long-axis view (**A**). However, this is not the most common situation encountered in clinical practice. (**B**) Wall-constrained proximal flow convergence with a oblong shape. This is frequently observed in case of posterior MV prolapse or commissural MR. There is a risk of over-estimation of MR EROA and RegVol by the PISA method. (**C**) Late-systolic MR. As shown in Doppler color coupled to M-mode, the MR is only present in the latter part of systole. Therefore the MR EROA which depends on a single time point measurement (the PISA radius) is not reliable to grade MR. (**D**) Multiple MR jets. The PISA method is not reliable to grade MR. Indirect quantitative methods should be considered. (**E**) Bi-leaflet prolapse with extensive MR from one commissure to another. The PISA method is inapplicable. Indirect quantitative methods should be considered. EROA, effective regurgitant orifice area; LV, left ventricular; MR, mitral regurgitation; MV, mitral valve; PISA, proximal isovelocity surface area.

The PISA method has been calibrated with LV angiography and TTE indirect quantitative methods (116–119). It is to date the most popular method among cardiologists to quantify MR. However, major limitations hamper its routine use in practice. All of these have been the object of many original studies, state-or-the-art reviews and editorials. Actually, the PISA method which has been first calibrated in experimental models relies on the core assumption that the flow would always be constant over time and orthogonal to well-defined hemispherical isovelocity shells until passing through a “pin-hole” orifice on a flat plane (120). However, these “idealized” conditions are rarely encountered *in vivo* in primary MR (Figure 6) (36, 37). The main limitations of the PISA method are summarized in Table 2. Those related to the measurement of PISA radius are numerous as recently illustrated by Hagendorff et al. (23). Interestingly, the vast majority of these issues had been well described since the very first PISA validation studies in the 1990s and have been given renewed attention in recent years thanks to the advances in 3D-echo imaging, comparison studies with CMR, software post-processing and computational models (45, 122–124). Therefore there are several features in primary MR where the PISA method may not be reliable including: wall-constrained eccentric jet (usually in case of posterior valve prolapse or commissural MR), multiple jets, late-systolic MR, or extensive Barlow's disease with bi-leaflet prolapse and diffuse MR leaking from one MV commissure to another (Figure 6).

Importantly, the mitral RegVol calculated by the PISA method is based on the EROA measured at a single time point during systole (125). However, in primary MR due to prolapse, the highest flow rate usually occurs during mid-to-late systole (38). Hence, the use of PISA does not truly fit physiological reality and can greatly over-estimate true RegVol. Theorically, both mitral EROA and RegVol should be integrated instantaneously over the entire cardiac cycle. Hence, the MR RegVol assessed by PISA only gives a rough estimate of the true RegVol.

*Main message for the clinician*

We suggest in routine practice to not report the mitral RegVol assessed by PISA. However, whenever it is possible to obtain a reliable measurement, without any of the clinical situations in primary MR as detailed above, the assessment of mitral EROA by PISA should be performed. Indeed, from an epidemiological perspective, each increase in EROA results in proportional higher risk of long-term mortality. Nonetheless, the numerous pitfalls in PISA method result in significant inter- and intra-observer variability on an individual basis. Hence, as suggested by other recent papers on the field, it is debatable whether PISA-based measurements should be considered as semi-quantitative rather than true quantitative parameters (23, 43).

#### 2.3.2. Indirect quantitative methods

##### 2.3.2.1. Pathophysiological basis

The indirect quantitative methods rely on the principle of conservation of mass: in a heart with normally functioning valves, the amount of blood crossing the MV (=transmitral volume) equals the amount of blood ejected into the aorta across the aortic valve. Hence, in patients with isolated MR, the LV total stroke volume (LVTSV) equals the aortic forward stroke volume (anterograde flow) plus the mitral RegVol (retrograde flow). Then, the MR RegFrac is obtained by dividing the MR RegVol by the LVTSV. Because they estimate MR RegVol and RegFrac without considering the MR regurgitant jet(s), the indirect quantitative methods are of great interest in case of very eccentric, multiple jets, or when the duration of MR is inconstant during systole, such as late-systolic MR, in other words all clinical situations where the PISA method is not reliable as detailed above. Certainly, they are the most appropriate approach for a thorough assessment of the MR severity from a hemodynamic perspective. However, they are only feasible in patients with sinus rhythm.

Noteworthy, the term of “volumetric methods” used in previous studies is somewhat misleading because indistinguishably referring to two different methods of calculating LVTSV: either using PW Doppler mode at the level of the mitral annulus to calculate the transmitral volume, or by computing the difference between LV end-diastolic (EDV) and end-systolic volumes (ESV) LVESV according to the 2D or 3D Simpson's method. For the sake of clarity, the terms of “PW Doppler quantitative method” and “volumetric method”, respectively referring to the former and the latter, will be used.

##### 2.3.2.2. PW Doppler quantitative method

**Figure 7:**
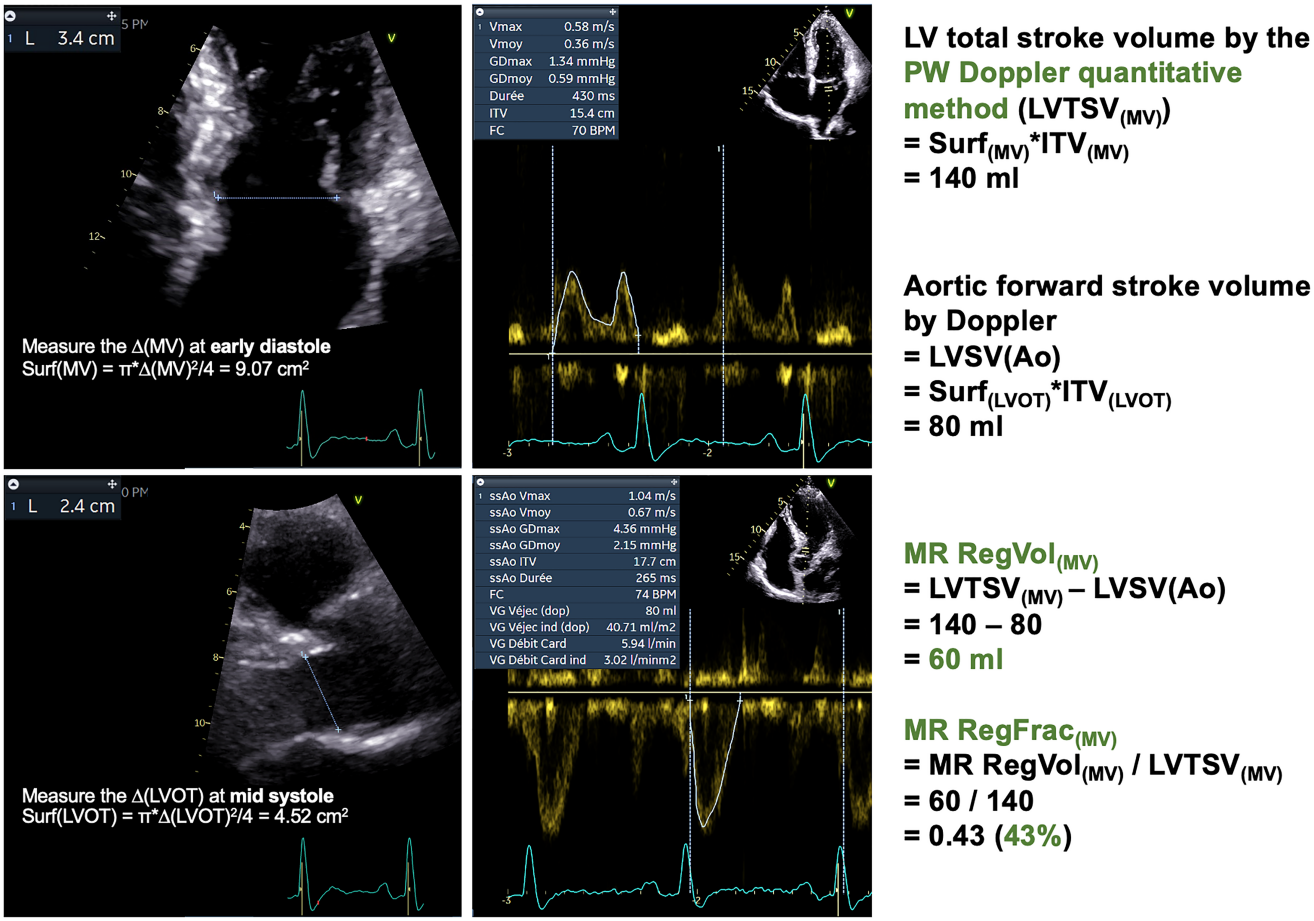
PW Doppler quantitative method. Same patient as Figure 5. The LV total stroke volume is computed by the Doppler method as follows: LVTSV_(MV)_ = π x (Δ_MV_)2 x VTI_MV_/4. Importantly, the *Δ*_MV_ must be measured at early diastole. Also the sample volume must be positioned at the level of the mitral annulus (not that of the leaflet tips). The aortic systolic forward stroke volume is computed by the Doppler method as follows: LVSV(Ao) = π x (Δ_LVOT_)2 x VTI_(LVOT)_/4. The *Δ*_LVOT_ must be measured at mid systole. The MR RegVol_(MV)_ is computed as the difference between the LVTSV_(MV)_ and the LVSV(Ao). Consequently, the MR RegFrac_(MV)_ is obtained by dividing the MR RegVol_(MV)_ by the LVTSV_(MV)_. *Δ*, diameter; LV, left ventricular/ventricle; LVOT, LV outflow tract; LVSV(Ao), LV systolic aortic forward stroke volume by Doppler method; LVTSV(_MV_), LV total stroke volume by the PW Doppler quantitative method; MR, mitral regurgitation; RegVol, regurgitant volume; RegFrac, regurgitant fraction; PW, Pulsed-Wave; VTI, velocity-time integral.

The PW Doppler quantitative method has been historically used to quantify MR (Figure 7) (126–128). Nowadays, it is not routinely used in practice. Indeed, this is a time-consuming approach. Then, this method suffers from two major sources of measurement errors: those related to the estimation of aortic forward systolic volume, as detailed thereafter, but also those related to the mitral annulus which significantly impact the estimation of transmitral volume (Table 2). The recent advances in imaging the mitral annulus with 3D-techniques may help to better estimate the transmitral volume but further research is required to validate this approach. The PW Doppler quantitative method is feasible only in patients with sinus rhythm, without aortic regurgitation but also without any degree of mitral stenosis or annular calcification. To date, this approach should not be used first-line but rather as an additional support when the assessment of other MR parameters leads to discrepant or inconclusive results.

##### 2.3.2.3. Volumetric method

**Figure 8:**
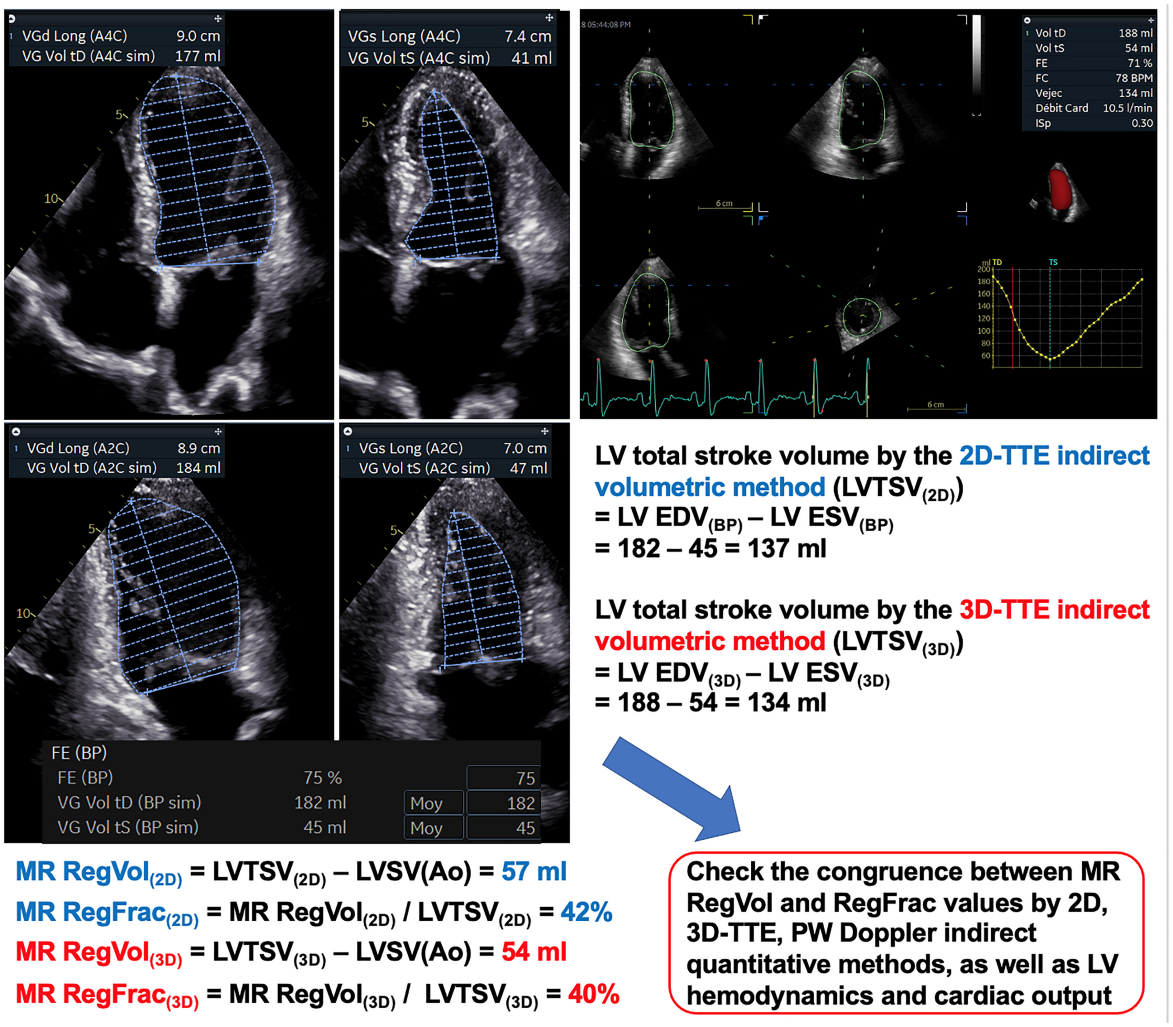
Indirect volumetric method. Same patient as Figures 5, 7. The LV total stroke volume is computed as the difference between LV end-diastolic and end-systolic volumes according to the 2D-biplane Simpson's method [LVTSV_(2D)_] or 3D-volumes [LVTSV_(3D)_]. The LV borders should be traced at the interface between the LV cavity and the compacted myocardium, and not at the blood-tissue interface (that is at the tip of the trabeculation). The aortic systolic forward stroke volume is computed by the Doppler method (see Figure 7). The MR-RegVol_(2D, 3D)_ is computed as the difference between the LVTSV_(2D, 3D)_ and the LVSV(Ao). The MR-RegFrac_(2D, 3D)_ is obtained by dividing the MR RegVol_(2D, 3D)_ by the LVTSV_(2D, 3D)._ The congruence between MR RegVol and RegFrac values obtained by the different indirect quantitative methods as well as LV hemodynamic and cardiac output should be carefully checked. Δ, diameter; BP, biplane; EDV, end-diastolic volume; ESV, end-systolic volume; LV, left ventricular/ventricle; LVSV(Ao), LV systolic aortic forward stroke volume by Doppler method; LVTSV_(2D)_, LV total stroke volume by 2D-indirect volumetric method; LVTSV_(3D)_, LV total stroke volume by 3D-indirect volumetric method; MR, mitral regurgitation; RegVol, regurgitant volume; RegFrac, regurgitant fraction.

The use of volumetric methods to quantify MR has been given renewed attention in recent years (Table 3). This is notably due to the intense debate surrounding the discrepant results between the COAPT and MITRA-FR trials which assessed the efficacy of transcatheter edge-to-edge repair for secondary MR (135, 136). Indeed several authors noticed inconsistencies when analyzing the reported echocardiographic data of the COAPT study (24, 25, 137, 138). Consequently, the need has arisen to reappraise the quantification of MR from a hemodynamic perspective (139, 140). The 2D-TTE volumetric method could suffer criticism because of two sources of measurement errors: those related to the LV volumes, and those related to the LV aortic forward stroke volume by PW Doppler. LV volumes measurements by 2D-TTE can be underestimated markedly compared to those obtained with CMR (141). The reasons explaining this include geometric assumptions of LV shape as elliptic according to the biplane Simpson's method, interobserver variability in the delineation of myocardial borders, apical foreshortening, or poor acoustic window (129, 142). Nonetheless, in our common experience it is possible to obtain reliable total and forward LV stroke volume values (and therefore to calculate MR RegVol and RegFrac) by 2D-TTE in the majority of patients when the image quality is sufficient (Figure 8) (130). This has recently been also reported by other groups (24, 43). To achieve this, the LV borders should be traced at the interface between the LV cavity and the compacted myocardium, and not at the blood-tissue interface (that is at the tip of the trabeculation) (143). The following tips can be helpful to obtain the most accurate apical views, without foreshortening the LV apex: (1) correctly position the patient; (2) select the acoustic window from the lowest rib space achievable; (3) ask the patient to breathe in/out as appropriate then to hold her/his breath while recording the apical views. The use of contrast echocardiography may help on a case-by-case basis but has not specifically been studied in the context of MR (144). Hence, after respecting an initial learning curve on normal exams (where the LVTSV should approximatively equal the aortic forward stroke volume), clinicians should be confident in their own volumetric measurements in pathological conditions such as MR.

**Table 3:** Relevant published studies in contemporary practice focusing on the volumetric method by echocardiography to quantify primary MR.

First author, year (Ref #)	Number of patients	Comparative method	Main findings
Cawley et al., 2013 (129)	26 with primary MR in sinus rhythm	– CMR volumetric method	– Good correlation between RegVol assessed by 2D-TTE versus CMR volumetric methods (*r* = 0.79). – Under-estimation of LV volumes assessed by 2D-TTE compared with CMR
Maréchaux et al., 2014 (130)	60 with primary MR due to prolapse (all grades) in sinus rhythm	– MR 4-grades 1+ to 4+ integrated approach	– High feasibility for determining 3D-TTE RegFrac (90% of patients) – Highly specific cut-off value of 3D-TTE RegFrac ≥ 40% to detect significant MR (3+ or 4+) – Large overlap zone for 3D-TTE RegFrac between 3 + and 4+ MR grades – Better discriminative value of 3D-TTE compared with 2D-TTE RegFrac between 1 and 2+ vs. 3–4+ MR grades
Heo et al., 2017 (131)	– 37 (validation group out of 152) who underwent CMR with primary (*n* = 27) or secondary (*n* = 10) MR (all grades)	– RegFrac by CMR volumetric method	– Moderate correlation (*r* = 0.53) with poor agreement (−75; 40 ml) between RegVol assessed by 2D-TTE versus CMR volumetric methods.
Levy et al., 2018 (132)	– 53 with primary MR due to prolapse (all grades)	– RegVol by CMR volumetric and 2D-PISA methods	– High feasibility (86% of patients) for determining 3D-TTE RegVol using automated fast 3D-TTE software (HeartModel) – Excellent inter- and intraobserver reproducibility for RegVol between 3D-TTE and CMR volumetric methods (56 ± 28 ml vs. 57 ± 23 ml) but significantly higher using 2D-PISA (69 ± 30 ml)
Lee et al., 2018 (133)	– 166 with severe primary MR due to chordae rupture in sinus rhythm	– RegVol by 2D-PISA	– Discordant MR severity grading found in 41% of patients using RegVol (>60 ml by 2D-PISA but < 60 ml by 2D-TTE volumetric method) – Moderate correlation (*r* = 0.53) with poor agreement (−25; 162 ml) between RegVol assessed by 2D-TTE volumetric versus 2D-PISA methods. – Small LV EDV or narrow PISA angle associated with over-estimation of RegVol by PISA versus 2D-TTE volumetric methods.
Altes et al., 2022 (134)	– 188 with moderate-to-severe or severe primary MR due to prolapse in sinus rhythm	– RegVol by 2D-PISA and CMR volumetric methods	– Weak correlation (*r *= 0.30) with poor agreement (–37; 63 ml) between RegVol assessed by 2D-TTE volumetric versus 2D-PISA methods. – Moderate correlation (*r* = 0.55) between RegVol assessed by 2D-TTE versus CMR volumetric methods – Fair correlation between LVEDV and RegVol (*r* = 0.68) but not RegFrac (*r* = 0.17) assessed by 2D-TTE volumetric methods

Nowadays, the 3D-TTE evaluation of LV volumes is part of the routine echo assessment in many centers. 3D-TTE has the advantages of not relying on geometric assumptions for volume calculations and of avoiding the need for foreshortened views. 3D-TTE-assessed LV volumes showed significantly less bias and lower intra- and inter-observer variability compared to CMR than those assessed with 2D TTE, at the exception of markedly enlarged LV volumes (145, 146). Importantly, no systematic difference in the measured LV ejection fraction between the 3D-TTE methods and CMR has been reported (147). Notwithstanding this, Levy et al. reported that in a cohort of patients with primary MR, while 3D-TTE LV EDV and ESV values were lower than those measured with CMR, LV TSV values were similar (bias: −5 ± 22 ml) (132). Consequently, MR RegVol values were similar between 3D-TTE and CMR (bias = −2 ± 12 ml). Maréchaux et al. found that in healthy patients without valvular disease 3D-TTE LV TSV and Doppler-based SV values were very close (bias −0.5 ml) without systematic bias. Therefore, MR RegFrac can be routinely determined using 3D-TTE with a high feasibility rate in patients with primary MR (130). However, this method requires good image quality and the benefits of 3D-TTE are tempered by its lower temporal resolution compared with 2D: a minimal frame rate of 25–30 fps should be reached to allow measurements. Also the reliability of 3D-TTE in patients with atrial fibrillation has not been specifically assessed in MR. Importantly, the echocardiography platform and analysis software used significantly affect the values of 3D-echo-determined LV parameters (148). Finally, one should not look for strict consistency between MR RegVol and RegFrac measurements by the volumetric method between 2D-TTE, 3D-TTE and CMR (if performed), but rather to ascertain that the values obtained are in the same range of MR grading severity.

Whichever LV volumes are measured using 2D- or 3D-TTE, the calculation of forward aortic stroke volume is the same and requires consideration. The potential causes for imprecise measurement of the LVOT diameter have been well documented in aortic stenosis (149). In patients with normal aortic valves, it is possible to obtain reliable estimates of forward aortic stroke volume when several conditions are fulfilled: proper positioning of the PW Doppler sample volume, good PW Doppler spectral envelope quality, adequate parasternal long-axis view with zoom on the LVOT, no aortic annular calcification, measurement of LVOT diameter at the level of aortic annulus in mid-systole (150). Nonetheless, the true shape of LVOT is not circular but rather elliptical, thereby resulting in stroke volume underestimation calculated using LVOT diameters vs. 3D LVOT areas (44, 151). Whether the estimation of forward stroke volume by the direct measurement of LVOT area by 3D-echocardiography would result in varying MR RegVol values needs further assessment.

*Main message for the clinician*

The indirect quantitative methods, feasible in patients in sinus rhythm, allow to obtain MR RegVol and RegFrac measurements, which correspond to hemodynamics. Therefore the 2D- and 3D-TTE volumetric methods should take a central role in the routine echocardiographic examination of patients with MR. The biggest LV volumes between those obtained by 2D vs. 3D-TTE should be selected to calculate LV total stroke volume. Whichever quantitative method is used, it is critical to look for consistency between LV, LA and mitral regurgitant volumes: for instance, it is not physiologically possible to have a MR RegVol value higher than the end-systolic LA volume. Also, the MR RegVol should not reach a value close or even higher than the LV total stroke volume, otherwise the “remaining” aortic systolic forward stroke volume would not be sufficient to ensure a cardiac output compatible with normal life. Both the PISA and volumetric methods have their own strengths and limitations in grading MR severity (152). To avoid mistaking the quantitative method actually used for a given measurement with the others, we suggest reporting PISA-assessed mitral EROA and volumetrically assessed mitral RegVol and RegFrac.

#### 2.3.3. Vena contracta area and proximal flow convergence assessed by three-dimensional echocardiography in primary MR

The integration of 3D echocardiography in modern-day echo units provides an opportunity to better appraise the complex geometry of the MV apparatus, leaflets and coaptation gap (153, 154). In particular 3D-echocardiography allows to consider the mitral EROA as a three-dimensional structure thereby not relying anymore on the many assumptions made when measuring 2D-VCW or 2D-PISA (155, 156). Several single-center cohort studies described good reproducibility of 3D-vena contracta area (VCA) or 3D-PISA with better agreement with CMR compared with two-dimensional measurements (124). The acquisition methods and technical considerations to acquire VCA and EROA by 3D-TTE have been well described in a recent review by Mantegazza et al. (52). Interestingly, these 3D methods may be of great interest to grade residual MR following transcatheter mitral valve repair (157, 158). In practice, although recent improvements in 3D-echocardiography techniques have made it possible to increase spatial and temporal resolutions, they still remain lower than with color 2D. Also the use of 3D-echo with Doppler color mode requires good image quality and sufficient framerate (>20 frames per second). Multibeat acquisition is helpful but not be feasible in patients unable to hold their breath or with arrhythmia. Thus, the subsequent MR jet analysis by multiplanar reconstruction (MPR) is time-consuming, implies advanced 3D-echocardiography skills and may be subject to interobserver variability. Therefore, the use of 3D-VCA and 3D-PISA currently remains limited to a few specialized echo units—although such limitations may be overcome in the near future by promising artificial intelligence (AI)-based tools. The availability of multiple-center prospective data for external validation of test-retest reproducibility and MR severity cut-off levels of 3D-VCA and 3D-PISA associated with adverse outcome would mark a major step towards more widespread use of these methods.

### 2.4. Conclusion on MR grading by echocardiography and work-up

At the end of echocardiographic evaluation, the MR should be classified as “significant”, “moderate”, “mild”, or “indeterminate”, as discussed in the following section (see Section 3.1). If there is no doubt that the MR is significant by 2D-TTE and with one or more indication(s) for MV surgery, then intervention should be planned promptly after Heart Team discussion according to MV repair probability, patient's risk profile and wishes. In this setting, the role of TEE is to detail the morphology of MV and mechanism(s) of MR either to guide the surgeon if a MV repair surgery is planned or to screen for the feasibility of percutaneous TEER.

If the MR is significant but symptoms are equivocal or absent and the patient is able to exercice, then either exercice stress echocardiography or cardiopulmonary exercice testing (CPET) are indicated to evaluate functional and hemodynamic tolerance of MR. Exercice stress echocardiography also allows to unmask exercice pulmonary hypertension at low workload, while CPET can detect reduced peak oxygen consumption related to MR consequences (159–161). Yet there is no established evidence that one of these two examinations would better stratify patients' risk than the other one in primary MR.

The work-up after TTE of patients with “moderate” or “indeterminate” MR is closely similar. Indeed the risk of patients with moderate MR is not uniform, as discussed further below. Several reasons can lead to classify MR as “indeterminate”. Discrepant results can be found between one or more MR specific echocardiographic parameters. Some of the MR parameters may not be feasible or reliable as previously detailed. Also MR should be graded as “indeterminate” if only mild or moderate apparently on the basis of MR echocardiographic parameters but with suspected valve-related symptoms, LA/LV enlargement or elevated pulmonary pressures without another explanation. Importantly, clinical situations or specific features of MR are at risk of misevaluation of severity including atrial fibrillation, late-systolic MR, bi-leaflet prolapse with multiple jets or extensive leak, wall-constrained eccentric jet or in women and/or older patients with complex MR mechanism. Then, a second-line examination (CMR or TEE) should be performed.

CMR should be considered rather than TEE as second-line imaging modality in patients in sinus rhythm, without contraindication for CMR and able to hold her/his breath, when the main remaining issue is to quantify MR. The comprehensive assessment of MR by CMR both in terms of quantification and arrhythmic risk stratification is beyond the scope of this review (162). The numerous advantages of CMR to quantify MR in patients are well-known (163). However, CMR comprises also some technical limitations which can lead to fluctuations in MR RegVol measurements by the indirect volumetric method. These include the choice of LV basal slice selected to estimate LV volumes because of partial-volume effects and through-plane motion. This is of particular concern in patients with prominent bi-leaflet prolapse (164). LV volumes estimates include the papillary muscles and trabeculae in the ventricular cavity. Then the forward stroke volume value (and thus MR RegVol) may vary regarding the location of through-plane phase contrast velocity mapping (165). Therefore, in our opinion, there is no need to oppose CMR and echocardiography: in experienced hands, both are excellent imaging modalities to assess MR severity and have their own strengths and pitfalls.

On the other hand, TEE should be preferred over CMR as second-line imaging after TTE when there is a specific need to comprehensively assess MV morphology in addition to grade MR severity. Also TEE is the second imaging modality of choice in patients with atrial fibrillation and/or with contraindication to CMR. The main complementary asset of TEE in primary MR evaluation is to provide a comprehensive evaluation of the mechanisms of the leak—notably thanks to high-resolution 3D imaging—and to predict probability of repair (52). MR grading by TEE requires specific considerations. TEE can provide better definition of 2D-VCW or proximal flow convergence than TTE thanks to its higher spatial and temporal resolution. Also the presence of an MR with Coandă phenomenon is well-defined by TEE. The CWD line can be aligned better with the direction of the jet using TEE, so the complete envelope of the signal can be obtained. All four pulmonary veins can be evaluated with TEE (100). 3D-VCA and 3D-PISA TEE provide substantially better image quality than TTE (156). However, for a same given RegVol, the MR jet can appear larger in TEE color Doppler mode because of the higher frequencies of the probe, or smaller in case of increased heart rate. Yet, while a close agreement has been reported between TTE and TEE MR quantitative values by PISA, the prognostic implications of cut-off values for MR EROA and RegVol by 2D-PISA have been studied only using TTE but not TEE (166). Importantly, MR is load-dependent, therefore its quantification may be influenced by the conditions under which TEE is performed (during surgery or under general anesthesia relative to light sedation) (167).

## 3. Discrepancies in echocardiography-based grading of MR severity

### 3.1. Considerations about MR grading

Disease grading according to severity is a cornerstone in medicine enabling clear communication between patients and practitioners and identification of appropriate therapies. Historically, the 4-grade classification system for MR severity arose from the qualitative findings of LV angiography used to assess MR (168). Enriquez-Sarano et al. first calibrated thresholds for MR quantitative measurements (EROA, RegVol, RegFrac) using LV angiography as the reference standard. The same group then subsequently demonstrated the prognostic value of these thresholds (116, 169). Thereafter, guidelines endorsed the 4-grade classification system resulting in its widespread use. Yet in routine practice it may be questioned whether this classification is truly discriminative for every patient with primary MR. Indeed Gammie et al. recently highlighted substantial variability in MR severity definition and reporting in contemporary clinical studies of mitral valve interventions (170). For example, 2+ MR was defined as moderate in 64% of studies, mild in 27%, and mild-moderate in 9%.

As such there is significant overlap between all MR semi-quantitative and quantitative parameters against LV angiography or PW Doppler quantitative evaluation. This can be explained in part by the sources of variability of each individual parameter, as well as by the choice of the gold standard. In particular there is considerable overlap between MR grades 3+ and 4+ (121, 167). Not surprisingly, the same overlap was found in recent studies evaluating the diagnostic accuracy of 3D-TTE parameters such as 3D-RegFrac (130). Actually, no one single echocardiographic parameter can discriminate with accuracy 3+ vs. 4+ MR for a given patient. Furthermore, prognosis worsens in asymptomatic MR 3 + patients over long-term follow-up until eventually reaching that of patients classified as MR 4 + at baseline (116). Antoine et al. demonstrated in a large study population that, compared with general population mortality, long-term excess mortality appears for moderate MR (EROA ≥ 20 mm^2^) and becomes notable at EROA ≥ 30 mm^2^ (117). They highlighted that patients with “moderate” MR according to guidelines (that is with RegVol between 30 and 60 ml) represent a very heterogenous group where the true challenge is to determine those who already suffer from consequences of MR and need intervention, those who may rapidly progress during follow-up and become eligible for MV surgery, and those at lower risk who should remain under active surveillance. As insightfully stated by Hung et al., the MR RegVol taken alone does not always capture the prognostic importance of MR, hence the patient's overall clinical status should be considered (171).

*Main message for the clinician*

The indication for MV surgery is discussed in heart teams for patients with 3+ and 4+ primary MR, based on the feasibility of MV repair, symptoms, other markers of adverse outcome in MR such as LV dysfunction, and operative risk. Considering the aforementioned, we think that numerical classification of MR should be abandoned. Following the latest guidelines, MR should be classified as “none”, “trace/mild”, “moderate” or “significant/severe”. Perhaps a further step would be to routinely prefer the term “significant” rather than “severe” MR to define the presence of a hemodynamically significant amount of regurgitant blood flowing across the MV. The term “severe”, implying by its etymology bad outcomes and requirements for intervention, should be employed in case of significant MR associated with one or several triggers for MV surgery. As our knowledge improves, it becomes clear that a rethinking of how we conceptualize MR grading is necessary. We should see it as a continuum integrating both quantification of MR and its consequences, even for patients with presumed “moderate” MR.

### 3.2. Discrepancies arising from the integrated multiparametric approach

The echocardiographic multiparametric approach has been precisely endorsed by guidelines to address the fact that no one single parameter can be used systematically to grade MR severity. However, the use of multiple parameters inherently results in potential discrepancies between one or more of them. Some of the reasons for this have been previously discussed: MR parameters may be impacted by technical settings, loading or other pathophysiological conditions, or echocardiographer skill. Two reproducibility studies reported only moderate interobserver agreement for grading MR by echocardiography (172, 173). Of note, both studies assessed reproducibility based on pre-recorded sets of echocardiographic loops and images. Actually, no prospective test-retest reproducibility study has been performed yet for echocardiographic grading of primary MR severity. Given the potential differences between echocardiographers when acquiring a MR proximal flow convergence or vena contracta, it can reasonably be hypothesized that doing so would result in greater interobserver variability. Moreover, some MR parameters are directly related to the amount of mitral regurgitant load (vena contracta, flow convergence area), whereas others are related to the impact of MR on heart chambers (LV, LA enlargement). This represents another source of discrepancy since MR volume and its consequences are not systematically related in a linear way.

Recently, a limited concordance between echocardiographic parameters of MR severity was reported, particularly for patients with more severe MR. On the other hand, the concordance between parameters was better when considering only 2D-VCW, MR EROA by PISA and RegVol (174). These findings correspond to the usual application of the integrated approach where quantitative MR parameters when measured prevail over the others. In another study from the same group, the strongest associations between MR RegVol by CMR and MR echocardiographic parameters were found for MR EROA, MR RegVol assessed by PISA, LVEDV, and flail leaflet, suggesting that these parameters should be weighted more heavily than others in echocardiographic grading of MR severity (175). A particular problem is that current guidelines do not suggest how discrepancies between multiple parameters should be handled, in particular whether the most severe parameter should be considered or if a consensus of parameters should be used. Therefore the latest ASE guidelines proposed hierarchical “weighting” of the different MR parameters in an algorithm to guide decision-making according to the presence or absence of these individual parameters and detailed for which combinations MR should be considered definitively mild or severe (27, 28). Gao et al. first investigated the ability of the ASE algorithm to rule in severe MR defined by CMR, proven post-operative LV reverse remodeling and improved functional class (176). Recently, Uretsky et al. observed in a subgroup of 48 patients who underwent MV intervention and post-operative CMR that severe MR by CMR was associated with LV reverse remodeling (defined as change in LVEDV after intervention), whereas “definitely severe MR” by the ASE algorithm was not (177). However, whether a significant decrease in LVDEV after MV intervention necessary implies that MR would be severe is uncertain. To date, data are scarce regarding the relationship between the echocardiographic integrated approach for MR grading or CMR-based MR parameters and clinical post-operative outcome in patients with primary MR.

*Main message for the clinician*

The large number of MR grading echocardiographic parameters offers the possibility to check the measured values regarding their congruence. The presence of incongruent findings should be explained by the MR type and features, loading conditions, acquisition techniques or echocardiographer skill. On the other hand, the measured values that appear demonstrably incongruent must be viewed critically and with caution. Hence, the large number of echocardiographic parameters available to grade MR offers both challenges and opportunities (178).

### 3.3. Discrepancies between quantitative MR parameters and outcome implications

Beyond the discrepancies between MR parameters, it is paramount to recognize that there are method-related differences of quantitative parameters such as MR RegVol. Indeed, mitral RegVols assessed by PISA or volumetric methods (either by 2D-TTE, 3D-TTE or CMR) can differ markedly (179). A recent meta-analysis of contemporary studies showed an overestimation with all 2D-echo measurements with only moderate agreement compared to CMR (124). Indeed, a major heterogeneity is observed for RegVol values between one or other method compared with CMR, with some patients having higher RegVol values by echo vs. CMR whereas other patients exhibit the opposite. Focusing on patients with at least moderate-to-severe primary MR exclusively due to prolapse (*n* = 188), we recently reported that the mitral RegVols obtained by PISA displayed poor correlation with those obtained with a volumetric method (CMR, TTE), thereby precluding direct comparison (134). In practice, this implies that one needs to account for these method-based differences and interpret RegVol according to different methods. Interestingly, Igata et al. recently observed that MR EROA and RegVol assessed by the volumetric method, but not by PISA, were predictive of outcome in patients with secondary MR and LVEF < 35% (180). Such a “head-to-head” comparative study of these MR quantitative methods by echocardiography remains to be done in primary MR.

Strikingly, the MR RegVol overestimation by PISA compared with volumetric methods has been reported to be more prevalent in patients with a normal or only mildly enlarged LV (133, 134). This has important implications for patients with small body surface area, notably women and/or elderly patients. Mantovani et al. observed smaller absolute cardiac dimensions and MR regurgitant volumes for women, thereby suggesting that these measurements should be indexed to body size (113). As a result, a given amount of MR RegVol is likely to impact differently heart chambers and hemodynamics (and thereby occurrence of symptoms) between two patients with different LV sizes and body surface areas. In practice, MR RegVol values obtained by volumetric methods below the 60 ml threshold retained by guidelines are frequently found for women and/or elderly patients, despite clear resolution of MR-related symptoms after intervention. Indeed, there is an expected proportional relationship between MR RegVol and LVEDV in primary MR. However, the LV response to MR may differ according to the underlying etiology (Barlow's disease, fibroelastic deficiency, or restrictive primary MR) or aging, because of impaired LV relaxation which may result in a lesser degree of MR RegVol and LV dilatation before symptom onset. Further studies are warranted to elucidate whether (1) sex-, age-, and/or MR etiology-specific thresholds could help to tailor the timing of intervention for these patients and (2) MR RegFrac rather than RegVol thresholds would allow better evaluation of MR severity, better prognostication and decision-making in all or selected subgroups of patients.

The main advantage of MR RegFrac is to consider the importance of MR from a more hemodynamic perspective, relating MR severity to the patient's LV dimensions and cardiac stroke volume. Using the volumetric method, MR RegVol is calculated using LVEDV. In the presence of normal LV volumes or only moderate dilatation, a unique 60 ml cut-off to define MR severity does not fit to all patients with primary MR. As reported by Uretsky et al., a third of patients with an MR RegVol assessed by CMR of less than 60 ml met the echocardiographic criteria for severe MR (175). Because MR RegVol and LVEDV (and thus LVTSV) are both related to body surface area, the calculation of MR RegFrac by the volumetric method could enable assessment of MR severity independently from LV size (134). Compared with RegVol, the clinical significance of MR RegFrac has been seldom studied, either on echocardiography or CMR. Maréchaux et al. reported that a cut-off of 3D-RegFrac ≥ 40% accurately discriminates patients with 3+ or 4+ MR, with a grey zone of overlap between 1+ or 2+ vs. 3+ or 4+ patients reduced by 3D-TTE compared to 2D-TTE (130). Consistently, CMR studies emphasized the discriminative value of a CMR-RegFrac ≥ 40%, that is below the 50% threshold still retained in guidelines, and with prognostic implications (181). Independently from the imaging modality used (2D-TTE, 3D-TTE, or CMR), further multicenter prospective studies should be conducted to standardize the severity thresholds of RegFrac based on outcome predictions in patients with primary MR.

*Main message for the clinician*

It is of upmost importance to conciliate MR quantitative measurements and hemodynamic findings when grading MR severity. If there are inconsistencies, either at individual patient- or study-level, the echocardiographic raw data should be carefully checked (26, 138). MR RegVol values estimated by PISA or indirect quantitative methods can differ markedly. Also, a unique MR RegVol cut-off of 60 ml to define MR severity does not fit to all patients with primary MR. MR RegFrac by the volumetric method relates the MR RegVol to the patient's LV dimensions and cardiac stroke volume. From a practical approach, a MR RegFrac assessed by the volumetric method of 40% or more is highly specific of significant (3+ or 4+) MR (182). Its routine calculation may allow to reconcile at least in part alleged discrepancies between echocardiography and CMR in grading MR.

### 3.4. Morphological features of primary MR at risk of discrepancies in grading MR severity

#### 3.4.1. Late-systolic MR

The presence of a “late-systolic MR” (that is with a jet largely predominating or exclusively present from mid to late-systole) and/or bi-leaflet impairment with mitral annulus disjunction should alert the echocardiographer to risk of misleading evaluation of its severity. Ten years ago, Topilsky et al. reported that the MR EROA assessed by PISA was differently linked to outcome in patients with mid-late systolic MR compared with those with holo-systolic MR (183). Indeed, for a same given EROA, patients with mid-late systolic MR had less harmful consequences of MR in terms of cardiac remodeling and outcome. Hence, the use of EROA alone to quantify MR severity should be avoided in such patients. However, the common belief that late-systolic MR would be rarely severe is untrue. Indeed, there is a risk of under-estimating MR severity using PISA in mid-late systolic MR notably due to hidden multiple jets, especially in case of bi-leaflet prolapse. Accordingly, we reported lower MR RegVol values assessed by PISA compared with volumetric methods in the presence of mitral annulus disjunction (134). A first reason explaining this would be that patients with Barlow's disease and mitral annular disjunction can display multiple jets. Another one would be that in addition to the MR regurgitant jet, these patients present with a “prolapsing volume”, that is a non-regurgitant blood volume shift resulting from the MV prolapse, localized between the mitral annulus and the leaflets in end-systole (184, 185). Levy et al. recently showed that this “prolapse volume” may significantly impact LVESV measurements and thus estimations of MR RegVol and RegFrac by volumetric methods (186). Whether the prolapse volume would have independent prognostic value in addition to the MR transvalvular load or is just a confounding factor when estimating MR RegVol is currently unknown.

It is crucial to acknowledge these clinical situations at risk of discrepancies in MR grading according to methods because they have prognostic implications. Penicka et al. demonstrated that multiple or late-systolic jets were major drivers of the discordance between PISA and volumetric methods (assessed by CMR) in asymptomatic patients with at least moderate primary MR (187). Furthermore, they showed that consideration of these discrepancies led to reclassification of these patients' risk on the basis of hard outcomes (all-cause mortality or indication for MV surgery). Indeed, patients with moderate MR by echocardiography but severe by CMR mainly displayed multiple MR jets and were at higher risk of adverse outcome during follow-up. Conversely, patients with severe MR by echocardiography but only moderate by CMR mainly exhibited late-systolic jets and shared better outcome. These important results highlight the fact that the indirect volumetric methods should be routinely performed in the presence of these features of MR at risk of discrepancies. In addition to Topilsky et al.'s findings, these data also underline the need for further research to discriminate among the mixed group of patients with mid-late systolic MR those who have moderate from those who actually have severe MR.

#### 3.4.2. Atrial fibrillation

Finally, the presence of atrial fibrillation (AF) at the time of echocardiography also substantially hampers MR severity grading. The variability of beat lengths, in particular when associated with tachycardia, impact the evaluation of Doppler color parameters such as VC or PISA. Also the LA can be enlarged because of the consequences of long-standing AF irrespective of the MR severity. Essayagh et al. established separate cut-off values to define LA dilatation in primary MR according to heart rhythm (sinus rhythm or AF) (13). AF particularly affects the accuracy and feasibility of volumetric methods and of 3D-echo imaging which may suffer from stitching artifacts. Patients with AF were present only in few studies assessing the diagnostic value of primary MR parameters. However, new-onset AF represents a turning point in the natural course of primary MR with a strong prognostic value leading to discuss intervention (188). Thus, the challenge is to discriminate patients with severe primary MR and new-onset AF who should be referred for MV surgery from those who still have moderate MR despite AF. In practice, whenever possible, it is better to re-assess a patient with AF after restoration of sinus rhythm by cardioversion.

*Main message for the clinician*

Late-systolic MR, multiple jets, bi-leaflet prolapse or atrial fibrillation are frequent situations at particular risk of misevaluation when quantifying MR.

### 3.5. Impact of discrepancies in MR severity grading on TTE serial assessment

Echocardiographic follow-up of patients with chronic primary MR is recommended at annual (moderate) or 6-month (severe) intervals (16, 17). Active surveillance performed in experienced centers for patients with truly asymptomatic severe primary MR is associated with a favorable prognosis, resulting in timely referral to MV surgery, and excellent long-term survival (189). Several practical points concerning TTE serial assessment of these patients are worth underlining. There are two questions being raised here: (1) Has the MR become severe or even worsened? (2) Did adverse cardiac remodeling due to MR occur or worsen?

The first question is easy to answer if the MR clearly worsened between two examinations (for instance moving from grade 2+ to 4+ due to sudden chordae rupture with new-onset flail leaflet). However, more progressive changes in chronic primary MR could be harder to detect. MR parameters depend upon machine settings or loading conditions so they may vary from one TTE to another independently from MR severity. The same machine with recorded settings should be used for the TTE follow-up of a given patient. Importantly, the echocardiographic images and loops should be re-examined side-by-side from one TTE examination to another to detect real changes not related to inter-observer variability. Doppler color MR jet is unable to assess MR progression (190). MR quantitative methods would theoretically allow better assessment of MR progression because they provide “objective” numerical values of MR grading. However, both PISA and volumetric methods may be impacted from intra- and inter-observer variability creating some “noise” in their ability to assess MR progression (191). Moraldo et al. purposefully demonstrated that relying on PISA alone to detect small changes in RegVol would require averaging a considerable number of beats (38). As they rightfully stated in their study, measuring several values and retaining the one which best fits the previous measured value as well as the clinical and full echocardiographic picture at the time of evaluation should be avoided. Because the 3D-TTE volumetric method has shown better reproducibility, it would arguably outperform other MR echocardiographic parameters in assessing MR progression, but this remains to be proven. For 3D-TTE, the same echocardiogram and software must be chosen to allow serial comparison of LV volumes (148). Finally, because of the discrepancies between RegVols assessed by PISA or volumetric methods, it is paramount to acknowledge that in any case measurements of RegVol by PISA or by 2D-TTE are interchangeable.

TTE serial assessment of patients with primary MR also intends to detect progression of LV diameters and volumes, LA size, degradation of LVEF or new-onset signs of pulmonary hypertension. Indeed, chronic MR may cause LA and LV dilatation and worsen mitral annular dilatation, thereby potentially damage the whole MV apparatus, which in turn results in further worsening MR severity and LV impairment (23). Great care should be taken in measuring LV and LA volumes from the same apical windows as previous TTEs. Again it is important to re-examine the echocardiographic loops side-by-side whenever needed. In practice, an apparently moderate MR but with progressive LV and/or LA enlargement without another explanation is suspect to have been under-estimated. Data are scarce on the prospective assessment of cardiac adaptations to primary MR over time. An increased mitral annulus size could be associated with a greater progression of MR severity (191, 192). In a community-based study, MR progression was associated with more severe ventricular and atrial remodeling and worse outcome (193). Latest ACC/AHA guidelines indicate that it is reasonable to consider MV surgery (class IIb, level of evidence C) in patients with severe primary MR and normal LV systolic function but with a progressive increase in LVESD approaching 40 mm or decrease in LVEF towards 60% on longitudinal follow-up (17).

*Main message for the clinician*

The echocardiographic longitudinal follow-up of patients with primary MR includes not only MR severity grading but also all its consequences on heart remodeling. Serial echocardiograms should be re-examined side-by-side. It is not rare in clinical practice to better appraise the MR severity on a second examination and eventually reclassify an initially “indeterminate” or “moderate” MR as significant.

## 4. Latest developments and future directions

Several advances in echocardiographic imaging and new approaches are under development to overcome current limitations of MR quantification methods. These include real-time automated 3D-PISA considering the variations of MR during systole (194, 195), automatic quantification of real-time 3D full-volume color Doppler (FVCD) TTE (131, 196), automated quantification of the density of the CWD MR signal, named the “average pixel intensity” method (197), and semi-automated MR quantification based on the Navier-Stokes equation with 3D color modeling of the velocity profile through the regurgitant orifice (198, 199).

All of these attempts to quantify MR with echocardiography in a semi-automatic way have shown positive results with better agreement with TEE or CMR than traditional methods, but only in single-center pilot studies with small sample-sized study populations. Also so far, most of these new MR quantification software products are vendor-dependent, thereby restraining their widespread use. Their incorporation into routine daily workflow will require external validation of their diagnostic yield as well as their implementation on echocardiographic machines. Lastly, the promise of AI-based innovative solutions in echocardiography can be expected to provide in the near future automatic quantification of valvular regurgitation (200).

## 5. Conclusion

Echocardiographic grading of primary MR severity relies upon an integrated multiparametric approach as recommended by the latest guidelines. It is of paramount importance that clinicians involved in valvular diseases are aware of the respective advantages and pitfalls of each of the different MR quantitative methods and imaging modalities used (2D, 3D-TTE, TEE). Appraisal of MR grading methods also requires comprehensive understanding of the basic principles of echocardiography hemodynamics and of the natural history of the disease. In particular it is important to acknowledge specific clinical situations at risk of misevaluation such as late-systolic MR, bi-leaflet prolapse with multiple jets, elderly patients and/or women, or presumed “moderate” MR but with valve-related symptoms and/or consequences on cardiac remodeling. Primary MR grading should be seen as a continuum integrating both quantification of MR and its consequences. The MR regurgitant fraction by indirect volumetric methods allows to appraise the MR from a hemodynamic perspective and should be the cornerstone of MR severity assessment. Advanced echocardiographic imaging techniques and progress in automatic measurements pave the way for new promising approaches to MR grading which need to be validated in clinical practice.
